# An engineered genetic circuit for lactose intolerance alleviation

**DOI:** 10.1186/s12915-021-01070-9

**Published:** 2021-07-05

**Authors:** Mingyue Cheng, Zhangyu Cheng, Yiyan Yu, Wangjie Liu, Ruihao Li, Zhenyi Guo, Jiyue Qin, Zhi Zeng, Lin Di, Yufeng Mo, Chunxiu Pan, Yuanhao Liang, Jinman Li, Yigang Tong, Yunjun Yan, Yi Zhan, Kang Ning

**Affiliations:** 1 College of Life Science and Technology, Huazhong University of Science and Technology, 430074 Wuhan, People’s Republic of China; 2 Innovation Base of Life Science and Technology, Qiming College, Huazhong University of Science and Technology, 430074 Wuhan, People’s Republic of China; 3 Key Laboratory of Molecular Biophysics of the Ministry of Education, Huazhong University of Science and Technology, 430074 Wuhan, People’s Republic of China; 4 State Key Laboratory of Pathogen and Biosecurity, Beijing Institute of Microbiology and Epidemiology, 100071 Beijing, People’s Republic of China; 5 Beijing Advanced Innovation Center for Soft Matter Science and Engineering (BAIC-SM), College of Life Science and Technology, Beijing University of Chemical Technology, Beijing, 100029 Beijing, People’s Republic of China

**Keywords:** Lactose intolerance, Genetic engineering, Synthetic biology, Gut microbiota, In vitro simulation, In vivo assessment

## Abstract

**Background:**

Lactose malabsorption occurs in around 68% of the world’s population, causing lactose intolerance (LI) symptoms, such as abdominal pain, bloating, and diarrhea. To alleviate LI, previous studies have mainly focused on strengthening intestinal β-galactosidase activity while neglecting the inconspicuous drop in the colon pH caused by the fermentation of non-hydrolyzed lactose by the gut microbes. A drop in colon pH will reduce the intestinal β-galactosidase activity and influence intestinal homeostasis.

**Results:**

Here, we synthesized a tri-stable-switch circuit equipped with high β-galactosidase activity and pH rescue ability. This circuit can switch in functionality between the expression of β-galactosidase and expression of L-lactate dehydrogenase in response to an intestinal lactose signal and intestinal pH signal, respectively. We confirmed that the circuit functionality was efficient in bacterial cultures at a range of pH levels, and in preventing a drop in pH and β-galactosidase activity after lactose administration to mice. An impact of the circuit on gut microbiota composition was also indicated.

**Conclusions:**

Due to its ability to flexibly adapt to environmental variation, in particular to stabilize colon pH and maintain β-galactosidase activity after lactose influx, the tri-stable-switch circuit can serve as a promising prototype for the relief of lactose intolerance.

**Supplementary Information:**

The online version contains supplementary material available at 10.1186/s12915-021-01070-9.

## Background

Lactose malabsorption, defined as the inefficient absorption of lactose, is reported to have a global prevalence of 68% until 2016 [1]. Symptoms of lactose intolerance (LI), defined as the presence of gastrointestinal symptoms caused by lactose malabsorption within the small intestine, occur when non-hydrolyzed lactose flows into the colon as a substrate for bacteria [1, 2]. This non-hydrolyzed lactose brings a high osmotic load into the colon luminal contents, which leads to increased water and electrolytes within the lumen, followed by stool softening, thus causing abdominal pain and cramps [3]. Additionally, lactose can be fermented into lactic acid and other short-chain fatty acids with gaseous products such as hydrogen, methane, and carbon dioxide, thus causing flatulence and diarrhea [3, 4].

The current treatments for LI mainly include dietary control, enzyme replacement therapy, and probiotic supplement. For dietary control, the moderation or restriction of lactose intake is recommended to relieve symptoms [5–7], which impacts people’s enjoyment of dairy products. Additionally, a recent study found that the administration of the highly purified short-chain galactooligosaccharide can help to adjust gut microbiota in order to improve LI [8]. Enzyme replacement therapy is another approach for individuals with LI [9]. Exogenous lactase intake may help lactose digestion and absorption for LI subjects, but its efficacy still lacks convincing evidence [2]. Compared to short-acting enzyme replacement, probiotic supplements have an advantage in their sustainability, [10] and a certain number of studies have confirmed that they can alleviate LI [11–13]. The key function of probiotics is to enhance the intestinal β-galactosidase (β-GAL) activity to aid in lactose digestion in the LI individual. Moreover, the endogenous β-GAL produced by the probiotics can persist more stably in the intestine. However, conventional bacteria cannot handle the pH drop caused by the fermentation lactose by the gut microbiota. The pH drop would cause physical discomfort, such as diarrhea, and most likely reduce the β-GAL activity [14, 15], thereby influencing the intestinal homeostasis.

Genetical engineering, which allows for precise control over a genomic sequence [16], might be the solution for the pH drop that affects non-modified bacteria. Current designs of engineered bacteria have been confirmed as effective for purposes such as infectious disease treatment [17] and cancer diagnostics [18], via synthetic biology. Moreover, engineered bacteria are believed to work more precisely and efficiently in addressing these diseases [19] as compared to wild-type bacteria. Previously, a recombinant strain expressing food-grade β-GAL for LI was constructed and evaluated [20, 21]. However, this engineered strain was still unable to handle the pH drop. As such, a stress-responsive system has the potential to make the bacteria more adaptable to the pH variation [22]. However, the influences of bacteria administration and pH drop on the gut microbiota remain unclear. These influences might be understood by observing gut microbiota variations during the lactose intake and bacteria administration phases.

In this study, we initially designed and constructed a tri-stable-switch circuit in the plasmid *pETDuet1-1* that had two functional states, which responded to signals of intestinal lactose and intestinal pH variation. Second, we transformed the circuit into the strain *Escherichia coli* BL21 to form the engineered strain BL21: *pETDuet1-1*, which was then used to confirm the functionality of the circuit in vitro and in vivo. Lastly, we investigated the variation of the murine gut microbiota and found that administration of the engineered strain BL21: *pETDuet1-1* was able to recover the gut microbiota of the mice affected by excess lactose intake.

## Results

### The tri-stable-switch circuit was designed to switch between two functionalities in response to environmental changes

**Fig. 1:**
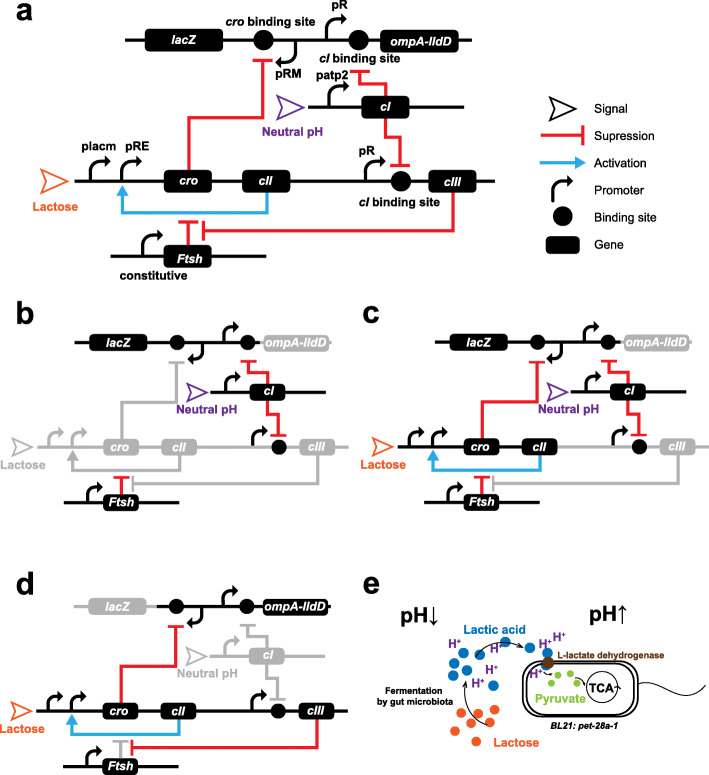
The tri-stable-switch circuit can switch between two functionalities in response to environmental change. **a** The design diagram of the tri-stable switch circuit. Parts of the circuit are derived from the bacteriophage *lambda*. The two promoters, *placm* and *patp2*, were selected to respond to the lactose and pH signals, respectively. The *placm* promoter is triggered by lactose, and the *patp2* promoter is weakened by the pH drop. **b** When BL21: *pETDuet1-1* colonizes the colon with a neutral pH, *lacZ* is stably expressed, and β-galactosidase (β-GAL) accumulates intracellularly. **c** When a flux of unabsorbed lactose occurs in the colon, the system switches to a transition state in response to lactose and pH signals. The expression of *ompA-lldD* for L-lactate dehydrogenase (L-LDH) is strengthened, and the expression of *lacZ* is weakened. **d** The system then focuses on the expression of *ompA-lldD*. **e** The fermentation of lactose by the gut microbiota causes a pH drop, while expression of L-LDH transforms lactic acid into pyruvate, thus recovering the pH. The pyruvate then permeates into the cell for the tricarboxylic acid (TCA) cycle

The tri-stable-switch circuit in the plasmid *pETDuet1-1* (Fig. 1a) was designed based on a tri-stable switch derived from the bacteriophage *lambda* [23]. The mutant lactose-inducible promoter *placm* (Additional file 1: Table S1) and pH-responsive promoter *patp2* (Additional file 1: Table S1) were cloned into this plasmid to sense the signals. The key enzymes applied within the system were the products of *lacZ* (β-galactosidase, β-GAL, Additional file 1: Table S1) and the fusion gene *ompA-lldD* (L-lactate dehydrogenase, L-LDH, Additional file 1: Table S1). The strain *E. coli* BL21 was chosen because it has been commonly used for stable expression of nontoxic exogenous proteins. The circuit was then transformed into *E. coli* BL21 to form the engineered strain BL21: *pETDuet1-1*. In theory, BL21: *pETDuet1-1* was able to dynamically switch between two functional states, which are regulated by a lactose signal and a pH signal. The theoretical working principle was as follows.

BL21: *pETDuet1-1* accumulated β-GAL after it colonized the colon (Fig. 1b). The average pH in the colon has been reported to be 7.0 [24], which, as a signal, maintained continuous *cI* gene (Additional file 1: Table S1) expression by inducing the *patp2* promoter. The expression of *cI*, which hindered the transcripts of the gene downstream of the pR promoter (Additional file 1: Table S1), suppressed expression of *ompA-lldD* gene and *cIII* gene (Additional file 1: Table S1), thus ceasing the function of the pH rescue. At this moment, the engineered bacteria would focus on the expression of the *lacZ* and accumulate β-GAL for supplementary lactose digestion when unabsorbed lactose fluxed into the colon.

BL21: *pETDuet1-1* gradually switched from *lacZ* expression to *ompA-lldD* expression after lactose fluxed into the colon (Fig. 1c). On the one hand, the lactose, as a signal, triggered the *placm* promoter, thus activating the positive feedback loop of pRE promoter, *cro* gene, and *cII* gene (Additional file 1: Table S1). The expression of *cro* then began to suppress *lacZ* expression after pRM promoter (Additional file 1: Table S1) via binding to its binding site [25]. Additionally, *cro* expression has been shown to be strengthened by *cII* expression, which is inhibited to a degree because *cII* expression is still suppressed by endogenous *Ftsh* gene expression [26]. On the other hand, fermentation of lactose by the gut microbiota has been demonstrated to produce lactic acid and other short-chain fatty acids, leading to a pH drop within the colon, which would weaken *patp2* and inhibit *cI* expression. However, previously expressed products of *cI* would continue to suppress the expression of *ompA-lldD* and *cIII* to a certain degree, and the suppression would gradually diminish as these products are degraded*.* Hence, *ompA-lldD* expression would gradually recover to a normal condition, producing a signal peptide [27], and L-LDH [28, 29] would be translocated to the cell membrane to convert lactic acid to pyruvate in the periplasm. Additionally, the gradual recovery of *cIII* expression would unsuppress *cII* expression by inhibiting endogenous expression of *Ftsh* [26]. Unsuppressed *cII* expression would then strengthen *cro* expression, thus accelerating the inhibition of *lacZ* expression. Once lactose fluxed into the colon, the entire system was in an intermediate state of double functions.

Once the switch process was completed, the BL21: *pETDuet1-1* focused on *ompA-lldD* expression (Fig. 1d), and the suppression of *cIII* and *ompA-lldD* expression was removed. Constitutive expression of *cIII* eliminated the suppression on *cII* expression via the endogenous expression of *Ftsh*, thus allowing the lactose-activated positive feedback loop to inhibit the *lacZ* expression. Expression of *ompA-lldD* continued to produce efficient signal peptides, allowing L-LDH to convert lactic acid to pyruvate in order to rescue the pH drop (Fig. 1e). Afterward, the pyruvate would be transported into the cell by its carrier protein [30, 31] for usage in the tricarboxylic acid (TCA) cycle [32]. Once the engineered bacteria complete digesting the lactose and restoring the pH, BL21: *pETDuet1-1* would subsequently switch to β-GAL accumulation until the next round of lactose ingestion. Thus, in this manner, BL21: *pETDuet1-1* would alternate its function in response to the lactose intake.

### The tri-stable-switch circuit was efficient under a range of pH conditions in vitro

**Fig. 2:**
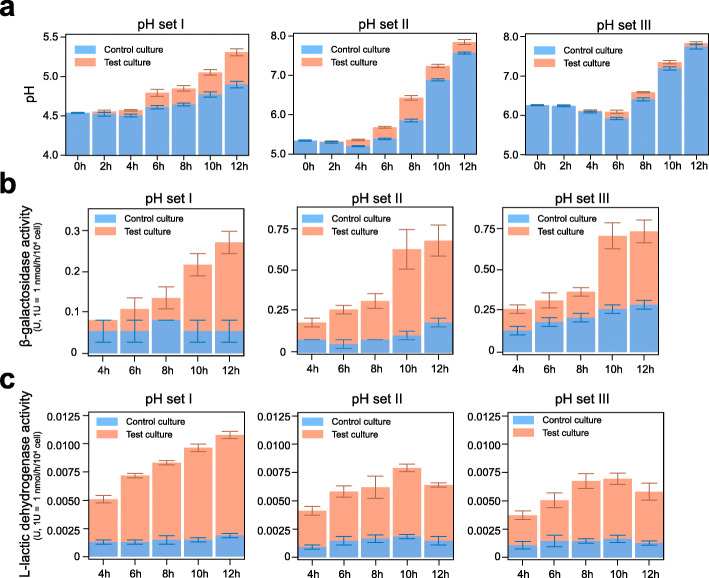
The tri-stable-switch circuit was efficient under a range of pH values in vitro. **a** The pH variation (mean ± S.E.M.) that occurred within bacterial cultures during 12 h of growth under different initial pH conditions. **b** The β-galactosidase (β-GAL) activity (mean ± S.E.M.) that occurred within bacterial cultures grown for 12 h under different initial pH conditions. **c** The L-lactate dehydrogenase (L-LDH) activity (mean ± S.E.M.) within bacterial cultures that were grown for 12 h under different initial pH conditions. In all panels, the control culture (BL21: *pETDuet1-0*) is colored in orange, while the test culture (BL21: *pETDuet1-1*) is colored in blue. All of the data were produced using three parallel experiments

The interactions between *cII* & pRE, *cI* & pR, *cro* & pRM, and *cIII* & *cII* have been tested using fluorescence detection (Additional file 1: Table S2, Additional file 1: Table S3, Additional file 2). The circuit switch was also confirmed to work in theory using mathematical simulation (Additional file 1: Table S4, Additional file 2). In order to test the circuit in vitro (Fig. 2), we prepared mediums with three pH values. In order to simulate the acidic conditions caused by excess lactose intake in the human colon, which typically has a pH of 7 [24], and the mouse colon, which normally has a pH of 5 [33], we adjusted the pH of these mediums by adding 0.1% lactic acid or 1% lactose. The three pH sets included pH set I (initial pH = 4.54 ± 0.012), pH set II (initial pH = 5.34 ± 0.02), and pH set III (initial pH = 6.25 ± 0.02).

We subsequently cultured various bacterial strains, including the test strain (BL21: *pETDuet1-1*, Additional file 3) and the control strain (BL21: *pETDuet1-0*) with an empty vector (Additional file 4) for 12 h in these three mediums and recorded the variation in the pH values and the expressed enzyme activity (Additional file 1: Table S5). As shown in Fig. 2a, the pH values of the control culture and the test culture began to increase at 6 h post-inoculation. The increase in the pH within the control culture was associated with two processes: (1) the metabolism of the substantial increase in the bacterial population, and (2) the consumption of the medium. However, the change in pH within the test culture was also dependent on a third process—expression of L-LDH, which helped facilitate the digestion of lactose and the pH increase. The increased pH caused by L-LDH was evident in pH set I. The pH of the test culture increased to a higher degree than that of the control culture (test culture: 4.54 ± 0.02 to 5.31 ± 0.075; control culture: 4.54 ± 0.01 to 4.9 ± 0.072). The pH increase in the test culture was also observed in pH sets II and III, but it was not as obvious as that in pH set I.

As shown in Fig. 2b and c, both the β-GAL activity and L-LDH activity, which were caused by the expression of the *lacZ* gene and *ompA-lldD* genes, respectively, in the circuit of BL21: *pETDuet1-1*, were higher in the test group as compared to the control group. Before 4 h, the enzyme activity measurements were unavailable because of the minimal amount of bacteria. After culturing for 4 h, the β-GAL activity of the test group continued to steadily increase in all three pH sets. In addition, 8–10 h post-inoculation, the β-GAL activity of the test group increased to the greatest extent and later flattened in pH sets II and III. Additionally, the L-LDH activity of the test group began to decrease in pH set II and pH set III 10 h post-inoculation. The corresponding pH range of the test group 8–10 h post-inoculation was 6.43 ± 0.10 to 7.23 ± 0.07 in pH set II and 6.58 ± 0.03 to 7.34 ± 0.07 in pH set III, which indicated that the dual-function switch of the circuit was completed for these pH ranges. These results suggested that relatively low pH values promote L-LDH expression in the circuit in order to remove the lactic acid to prevent the increase in pH. The increased pH then makes the circuit begin to switch gradually from L-LDH expression to β-GAL expression, which would continue until the pH is close to neutral.

### The tri-stable-switch circuit helped mice to recover the pH drop caused by excess lactose intake

**Fig. 3:**
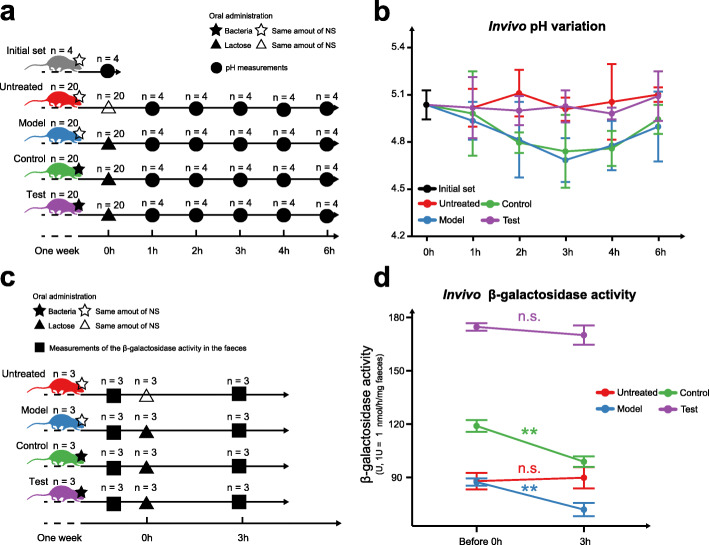
The tri-stable-switch circuit helped mice recover from the pH drop caused by excess lactose intake. **a** Five groups of mice: (1) initial set (n = 4), (2) untreated group (n = 20), (3) model group (n = 20), (4) control group (n = 20), and (5) test group (n = 20) were subjected to different operations in the first week. Mice in the control group and the test group were administrated with bacteria (BL21: *pETDuet1-0* in the Control group, BL21: *pETDuet1-1* in the test group; OD_600_ = 1) in a total volume of 0.3 mL of a 0.9% normal saline (NS) suspension daily. The other groups were administrated daily with the same volume of 0.9% NS. At the time point of 0 h, mice in the initial set were euthanized for pH measurements. The mice of the model, control, and test group were administrated the lactose solution (12 mg of lactose per 20 g of body weight), and the mice of the untreated group were administrated the same volume of 0.9% NS. During the following 6 h, four mice from each group were euthanized at each time point for pH measurements. **b** The pH variation (mean ± S.D.) of the mouse colons during 6 h. The initial set is designated as the initial point of four other groups. The pH variation of different groups is colored differently. The pH variation of the model and control group is statistically significant (*P* < 0.05, Student’s *t* test, Additional file 1: Table S6). **c** Four groups of mice: (1) untreated group (n = 20), (2) model group (n = 20), (3) control group (n = 20), and (4) test group (n = 20) were subjected to different operations in the first week, as described in **a**. Before the time point of 0 h, mice of all the groups were stimulated to defecate for measurements of β-galactosidase activity. At the time point of 0 h, mice of the model, control, and test group were administrated the lactose solution (12 mg of lactose per 20 g of body weight), and mice of the untreated group were administrated the same volume of 0.9% NS. At the time point of 3 h, mice of all the groups were stimulated to defecate for measurements of β-galactosidase activity. **d** The β-galactosidase activity variation (mean ± S.D.) of the mouse feces during 6 h. The variation of different groups is colored differently. * P < 0.05, ** P < 0.01; n.s., not significant. Student’s *t* test

The in vitro experiments confirmed the theoretical feasibility of the tri-stable-switch circuit to alleviate LI by switching between β-GAL expression and L-LDH expression, but whether it could work in vivo remained unclear. We thus divided 84 mice into five groups, including (1) initial set (n = 4), (2) untreated group (n = 20), (3) model group (n = 20), (4) control group (n = 20), and (5) test group (n = 20) in order to investigate how the circuit functioned in vivo. As shown in Fig. 3a, mice in the control and test groups were administrated bacteria (BL21: *pETDuet1-0* in the control group and BL21: *pETDuet1-1* in the test group; OD_600_ = 1) in a total volume of 0.3 mL in a 0.9% NS suspension daily during the first week. The bacteria were confirmed to colonize the colons of the mice, which lasted for at least 24 h (Additional file 2). The other groups were given the same volume of normal saline (NS) daily. The pH of the colons of the mice in the initial set was set as the pH value at 0 h for all groups. At the time point of 0 h, mice of the model, control, and test groups were administrated the lactose solution (12 mg of lactose per 20 g of body weight), and mice of the untreated group were administrated the same volume of 0.9% NS. The pH values of the colons of the mice in the remaining four groups were then tested at each time point (Additional file 1: Table S6) and graphed to illustrate the variation in pH (Fig. 3b). The colon pH of the model group and control group decreased to 4.66 ± 0.15 and 4.72 ± 0.25, respectively, from 0 h to 3 h, and then recovered to 4.89 ± 0.24 and 4.94 ± 0.1, respectively, from 3 h to 6 h. However, the colon pH value of the untreated group without lactose intake and the colon pH of the test group with BL21: *pETDuet1-1* were relatively stable. Thus, these results indicated that the tri-stable-switch circuit prevents the pH drop in mouse colons caused by an excessive intake of lactose, thereby restoring intestinal homeostasis and relieving LI.

Moreover, we then tested fecal β-GAL activity using another set of the four groups of mice, including (1) untreated group (n = 3), (2) model group (n = 3), (3) control group (n = 3), and (4) test group (n = 3). The operations in the first week and at the time point of 0 h were the same as the operations described above (Fig. 3c). We then tested the β-GAL activity in the feces of mice before the time point of 0 h, and at the time point of 3 h (Additional file 1: Table S7, Fig. 3d). The β-GAL activity of the test group was found kept at a high and stable level (*P* = 0.27, Student’s *t* test), suggesting that the colonization of the BL21: *pETDuet1-1* has prepared enough β-GAL activity for the following lactose intake. No evident variation in the β-GAL activity of the untreated group was observed as well (*P* = 0.68, Student’s *t* test). Nevertheless, the β-GAL activity of the model and control groups significantly decreased at 3 h (model group: *P* = 0.0072, control group: *P* = 0.0015, Student’s *t* test). These results indicated that the colon pH drop might decrease the intestinal β-GAL activity, and the tri-stable-switch circuit could keep intestinal pH stability and high intestinal β-GAL activity.

### The tri-stable-switch circuit helped the murine gut microbiota recover from the effects of excessive lactose intake

**Fig. 4:**
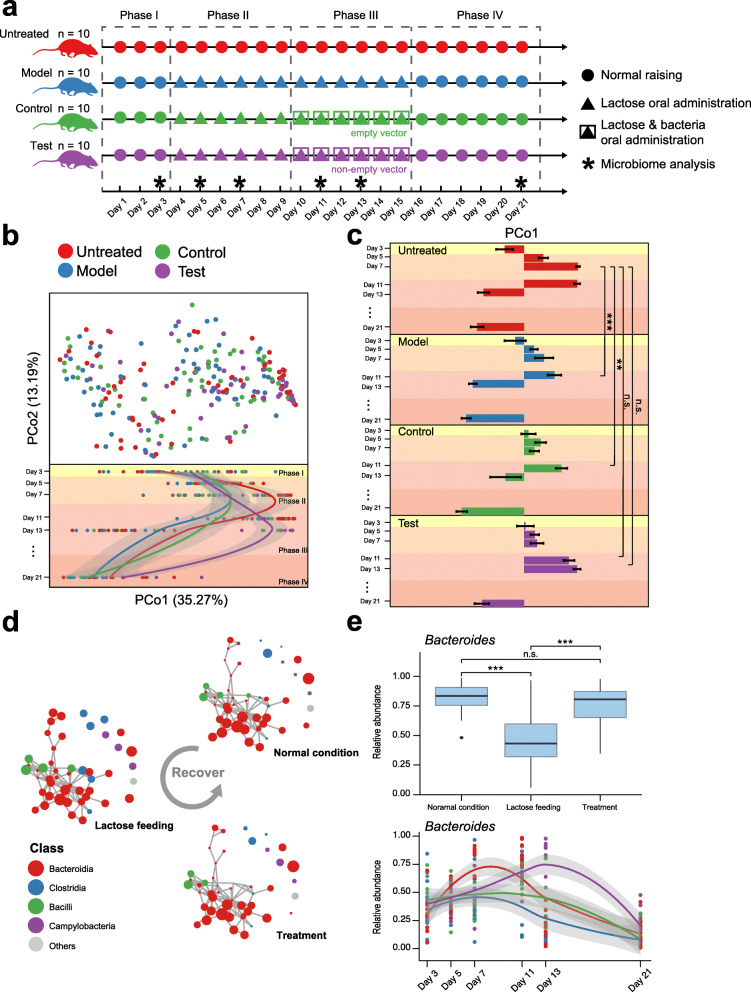
The tri-stable-switch circuit helped the gut microbiota of mice recover from the effects of excess lactose intake. **a** The design of the murine experiment for gut microbiota profiling. **b** Top panel: The gut microbiota composition of individual mice in the untreated group (n = 58), model group (n = 59), control group (n = 53), and test group (n = 55) plotted on a principal coordinate analysis (PCoA) using Jensen-Shannon Divergence (JSD). Bottom panel: The gut microbiota samples are plotted according to their collection date on the y-axis over 21 days, and their position on the x-axis is plotted according to their first principal coordinate in the JSD PCoA. A Loess regression is applied to these points using the collection date and principal coordinate 1 (PCo1) coordinates, and the curves are plotted in different colors according to their groups, with the 95% pointwise confidence interval band shaded gray. **c** The PCo1 coordinates (mean ± S.E.M.) from the four trial groups across 21 days. * P < 0.05, ** P < 0.01, *** P < 0.001; n.s., not significant. Mann-Whitney-Wilcoxon test. **d** The network was constructed using unweighted adjacency matrices produced by SPIEC-EASI. Sparse inverse covariance estimation based on glasso determined whether there was a correlation in abundance between the two amplicon sequence variants (ASVs), indicated by the gray edge. The size of the node represents the mean abundance calculated using different subsets of samples (defined in the “Results” section and “Methods” section). The color of the node represents the class level that the ASV is classified as. **e** Top panel: The boxplot displays the differences in *Bacteroides* abundance among the three data subsets in **d**. *** P < 0.001; n.s., not significant. Mann-Whitney-Wilcoxon test. Bottom panel: Each of the points represents the *Bacteroides* abundance of one sample from the four groups defined in **a**. A Loess regression was applied to these points, and the curves are plotted in different colors according to their groups, with the 95% pointwise confidence interval band shaded gray

In order to understand the effects of the engineered bacteria on the murine gut microbiota, we conducted a time-series experiment using a high-frequency sampling of mice fecal samples (Additional file 1: Table S8). As shown in Fig. 4a, four groups of mice (i.e., untreated group, model group, control group, and test group) were subjected to different interventions. The experiment lasted for 21 days and was divided into the four phases: normal care (Phase I), lactose challenge (Phase II), bacterial treatment (Phase III), and restoration (Phase IV). For Phase I, during which the four groups received normal care, the objective was to stabilize the physical signs and the gut microbiota of the mice in the four groups. For Phase II, during which lactose was fed to the model, control, and test groups, the objective was to investigate the influence of excess lactose on the gut microbiota. Phase III, in which BL21: *pETDuet1-1* was fed to the test group while empty-vector-containing BL21: *pETDuet1-0* was fed to mice in the control group, was used to determine whether BL21: *pETDuet1-1* can alleviate LI. In Phase IV, we intended to observe whether the bacteria caused any side effects in the host mice.

The dynamics of the mice gut microbiota differed among the four groups over the 21-day trial. From days 3 to 11, most of the gut microbiota samples from the Untreated group trended toward the right end of the principle coordinate 1 (PCo1) axis, whose degree was more considerable than those of the mouse groups administrated with lactose (Fig. 4b, c). In other words, excessive lactose intake inhibited the shift in the microbiota toward the right end of the PCo1 axis during this period, which began to be obvious at day 7. Nevertheless, among lactose-affected gut microbiotas, only those of the test group arrived at the same degree as those of the untreated group (Figs. 4b, c) after a time lag. Thus, it appeared as though the engineered bacteria were able to weaken some restrictive effects of the lactose.

We then constructed an unweighted-glasso network based on amplicon sequence variants (ASVs). To figure out the exact taxa affected by the inhibitory effects, we calculated the mean abundances of the top 50 most abundant ASVs in the network using samples from three data subsets including “normal condition,” “lactose feeding,” and “treatment” (Fig. 4d, Additional file 1: Table S8). We used samples of the untreated group at days 7 and 11 for the network of “normal condition” because they were most prominent characteristics in the normal stage (Fig. 4c). We used the samples from the model and control at days 5, 7, and 11 and test groups at days 5 and 7 for the network of “lactose feeding” because these samples were under the effects of lactose. We used the samples from the test group at days 11 and 13 for network of “treatment” because these samples have been treated with engineered bacteria. The networks showed that the microbial patterns would be largely affected by lactose intake and then recovered to the pattern that was similar to the original normal pattern after bacterial treatment (Fig. 4d).

Moreover, we found that 35 out of 50 most abundant ASVs in the networks were classified as class Bacteroidia (Additional file 1: Table S9), and genus *Bacteroides* was the most common genus in the altered microbiota and whose altered abundance was highly accordant with the microbiota variation against PCo1 (Fig. 4c, e). As compared to the “normal condition” network, 29 out of 50 most abundant ASVs were differentially abundant in the “lactose feeding” network, but had an abundance similar to the “treatment” network. For instance, an ASV that is most likely *Bacteroides acidifaciens* (confidence: 0.90) had an abundance of 0.285 ± 0.036 in the “normal condition” network and a similar abundance of 0.282 ± 0.037 in the “treatment” network, but it had a decreased abundance of 0.199 ± 0.016 in the “lactose feeding” network (*P* = 0.027, Wilcox test). Other ASVs that were also most likely *Bacteroides acidifaciens* were found to have similar distributions (Additional file 1: Table S9). Additionally, an ASV that is most likely *Lactobacillus murinus* (confidence 0.92) had increased abundance in the “lactose feeding” network as compared to that in “normal condition” network (*P* = 0.001, Wilcox test), while there was no significant difference in its abundance found between the “normal condition” network and the “treatment” network. These results indicated that excessive intake of lactose might inhibit the growth of the genus *Bacteroides* in the gut microbiota of mice during Phase II, while the administration of BL21: *pETDuet1-1* removed this inhibition, such that the murine gut microbiota proceeded to the variation in Phase III, similar to the gut microbiota of the untreated mice in Phase II.

## Discussion

In this study, we designed a tri-stable-switch circuit with the ability of β-GAL accumulation and pH rescue. The engineered bacteria equipped with this circuit can flexibly adapt to the variation of the intestinal environment, thus timely digesting lactose and rescuing the intestinal pH drop, along restoring the gut microbiota. We believe using engineered bacteria equipped with this tri-stable-switch circuit can serve as a promising method for LI.

The tri-stable-switch circuit compensates for the defect of non-modified bacteria by digesting lactose and enabling an additional function of pH rescue. The pH drop caused by the fermentation of lactose by the gut microbiota causes diarrhea and reduces the activity of the intestinal β-GAL and disrupts intestinal homeostasis. Therefore, the tri-stable-switch circuit was designed to respond to the signals of pH and lactose concentration and then dynamically switch between two functional states: accumulation of β-GAL and pH rescue. The accumulation of β-GAL facilitates the digestion of lactose with high efficiency, while the pH rescue function maintains intestinal homeostasis, thereby giving the bacteria engineered with this circuit better adaptability to the intestinal environment than non-modified bacteria. These two functions have been confirmed in our in vitro and in vivo assays.

Interestingly, in a 21-day in vivo mouse experiment, the engineered bacteria with the tri-stable-switch circuit might recover the murine gut microbiota from the effects of excessive lactose intake. Once lactose flowed into the colon, the fermentation of the unabsorbed lactose by the native gut microbiota would produce acids and gas, which resulted in a pH drop in the colon. This environmental change within the colon would, in turn, influence the native gut microbiota. However, colonization of the engineered bacteria would influence the native microbiota through microbial interactions, and the expression of β-GAL and L-LDH would also influence the intestinal environment. We found that the normal variation within the intestinal microbiota was affected by the excessive intake of lactose and found the lactose effects were eliminated to a certain degree by the engineered bacteria. The microbe most influenced by this process was *Bacteroides acidifaciens*, whose growth was previously reported to prevent obesity and improve insulin sensitivity in mice [34]. This study provides a reservoir of the influenced microbes, further investigations on which are warranted in future.

This study has limitations. First, this study mainly underscores the design of the tri-stable-switch circuit and the confirmation of its functionality. Hence, for this purpose, the *E. coli* BL21 strain would be more suitable for confirmation of the functionality of a prototype rather than for therapeutic intention. To apply this tri-stable-switch circuit to humans still needs more sophisticated studies in order to determine a proper chassis and ensure safety. Second, it is already known that the pH of the mice colon is lower than that of the human colon. The switch of the tri-stable-switch circuit was engineered to complete its functions in a pH range close to a neutral condition, which better fits in the human intestine. Nevertheless, we observed in the in vitro experiments using a broad range of pH conditions, the tri-stable-switch circuit still works well at a lower pH. Third, it was a missed chance for us to investigate on the variation of the absolute bacterial amount, which might help to characterize the lactose induced dysbiosis. Forth, we are not able to explain why the gut microbiota of mice in the untreated group was unstable. However, we have shown that the microbiota can be affected by an excessive intake of lactose and restored via administration of bacteria engineered with the tri-stable-switch circuit.

## Conclusions

In summary, the tri-stable-switch circuit can serve as a promising prototype for LI symptoms relief, especially by flexibly adapting to environmental variation, stabilizing colon pH, and maintaining β-galactosidase activity after lactose influx.

## Methods

### Experimental design

This study aimed to construct a genetic circuit with a tri-stable switch. To test the functionality of the circuit, we used a 12-h in vitro simulation and a 6-h in vivo experiment to verify that the circuit works in lactose digestion and pH adjustment. We also used a 21-day in vivo model to monitor the variation of the murine gut microbiota under the influence of the circuit.

### Circuit construction

Sequences were synthesized by Integrated DNA Technologies (IDT, Coralville, IA, USA) as PCR templates. The 3A assembly was based on isocaudomer digestion and ligation. Forward primers included prefix 5′-GAATTCGCGGCCGCTTCTAG-3′ for the coding sequence and 5′-GAATTCGCGGCCGCTTCTAGAG-3′ for the non-coding sequence. Reverse primers contained the suffix 5′-TACTAGTAGCGGCCGCTGCAG-3′. Then the prefix and suffix were added to the DNA segments through PCR (Additional file 5: Table S10). The double enzyme reaction was then performed on these DNA products (Additional file 5: Table S11). The *pSB1C3* backbone and target DNA segments with sticky ends were assembled together by T4 ligase (Additional file 5: Table S12). In-Fusion was then used to assemble the functional circuits with the 3A-assembled intermediate parts. The 5′ end of the In-Fusion primer contained 15 bases that were homologous to 15 bases at the end of the DNA fragment it joined. The system was incubated (Additional file 5: Table S13) for 15 min at 50 °C, and it was then placed on ice.

### Transformation

All cloning plasmids (with the *pBC1C3* backbone) were transformed into *E. coli DH5α* cells by the standard competent cell transformation method [35]. The strains were cultivated on Luria-Bertani (LB) plates (1% tryptone, 0.5% yeast extract, 1% NaCl, and 1% agar) at 37 °C. For positive selection, ampicillin (1%) was applied to the medium*.* The complete parts were cloned into the *pETDuet-1* backbone by inserting it into the PstI site. The extracted plasmids (with the *pETDuet-1* backbone containing the T7 promoter conserved in *E. coli DH5α*, Additional file 3) were transformed into *E. coli* BL21 for gene expression. *E. coli* BL21 encodes a native β-galactosidase, but does not encode a native L-lactate dehydrogenase. Thus, *E. coli* BL21, which was transformed with an empty plasmid (*pETDuet-0*, Additional file 4), was used as a control for the other experiments.

### Fluorescence detection

First, 0.2% (volume) of activated bacteria containing constructed circuit and their respective control was inoculated into M9 medium (Additional file 5: Table S14) and cultured overnight in a 37 °C shake incubator. Bacteria without the pathways were set as a blank control. Then 10% (volume) of the bacterial culture was inoculated into fresh medium and cultured to an OD600 of 0.6. The bacterial culture was then diluted with the corresponding medium to an OD600 of 0.1, and isopropyl β-D-thiogalactopyranoside (IPTG) (1 mg/mL) was added into the culture. The culture was incubated continuously at 37 °C in the dark. Every hour, a 100-μL aliquot of the culture for each sample was added into a black 96-well plate to measure both the fluorescence and OD_600_ (Additional file 5: Table S15). Before measurement, the plate was covered with tin foil.

### 12-h in vitro simulation

The Luria-Bertani (LB) broth medium was firstly made and sterilized. The 0.1% sterilized lactic acid and 1% sterilized lactose were then added to the LB medium. The adding of lactic acid and lactose was used to simulate the case that the excessive lactose flowed into the colon, part of which was then fermented to lactic acid by gut microbiota. To simulate the environmental pH of the mice colon and human colon, we used 1 M sterilized NaOH and 1 M sterilized HCl to adjust the pH values of the LB mediums to three pH sets, including the pH set I (initial pH = 4.54 ± 0.012), the pH set II (initial pH = 5.34 ± 0.02), and the pH set III (initial pH = 6.25 ± 0.02). Then, 1% ampicillin was added to eliminate contamination. The control strain BL21: *pETDuet1-0* and the test strain BL21: *pETDuet1-1* were then inoculated into the three pH sets with three replicates at initial OD_600_ of 2.038 and 2.081. During the 12 h in vitro culturing, 2 ml of the culture was taken out every 2 h at the clean bench to measure the pH value and the enzyme activity. The β-galactosidase activity was determined by the colour reaction of p-nitrophenol using “β-galactosidase activity test kit” (Solarbio, Cat. No. BC2585, Beijing, China). The l-lactate dehydrogenase activity was determined by pyruvate’s colour reaction using “l-lactate dehydrogenase (L-LDH) activity test kit” (Solarbio, Cat. No BC0685, Beijing, China).

### 6-h in vivo experiment

The 6-h in vivo experiment was conducted on post-weaning BALB/c mice, which was demonstrated and used as the LI model mice in previous studies [20]. Male BALB/c mice from animal care aged 7~8 weeks were used for the trial. Before the test, mice were housed under specific pathogen-free (SPF) conditions with a 12-h light/dark cycle (8 AM–8 PM for light, 8 PM–8 AM next day for dark) and without food restriction for 1 week. There were four replicates for each mice group (initial set, untreated, model, control, test) at each time point. In the first week, mice in the control group and the test group were daily administrated with bacteria (BL21: *pETDuet1-0* for the control group, BL21: *pETDuet1-1* for the Test group. OD_600_ = 1) in a total volume of 0.3 mL 0.9% NS suspension. Other three groups were daily administrated with the same volume of 0.9% NS. At the time point of 0 h, mice of the initial set were killed to test colon pH values as a reference, and mice of other four groups were administrated with the lactose solution (12 mg of lactose per 20 g of body weight). In the following 6 h, four mice of each group were killed at each time point (1 h, 2 h, 3 h, 4 h, 6 h) for pH measurements. The whole mice colon contents were removed and mixed, and the pH was determined using a pre-calibrated pH FE28-Meter (Mettler-Toledo). pH measurements were taken three times with the colon contents being re-mixed, the pH meter being washed with distilled water, and the calibration checked between measurements.

Another set of the four groups of mice was used to test fecal β-GAL activity, including (1) untreated group (n = 3), (2) model group (n = 3), (3) control group (n = 3), and (4) test group (n = 3). The operations in the first week and at the time point of 0 h were the same as the operations described above. The mice feces were collected by stimulated defecation. The collected feces were washed with the phosphate buffer saline (PBS) for three times and suspended with the PBS (mass of feces: volume of PBS = 1:9). The suspension liquid was then crushed with ultrasound and centrifuged at 5000×g for 10 min. The supernatant was taken for testing β-GAL activity, determined by the colour reaction of p-nitrophenol using β-galactosidase (β-GAL) activity test kit (Solarbio, Cat. No. BC2585, Beijing, China).

### 21-day in vivo model

The 21-day in vivo model was conducted on post-weaning BALB/c mice. Male BALB/c mice from animal care aged 7~8 weeks were used for the trial. Before the test, mice were housed under specific pathogen-free (SPF) conditions with a 12-h light/dark cycle (8 AM–8 PM for light, 8 PM–8 AM next day for dark) and a normal diet for 1 week. Ten replicates of each group (untreated, model, control, and test) were identified by pierced ear labels and separate cages (Additional file 1: Table S8). The trial was divided into the following 4 phases: Phase I: 3 days of normal conditions; phase II: 6 days of lactose challenge; Phase III: 6 days of bacterial treatment; and Phase IV: 6 days of restoration. The four groups of mice were treated differently during the four phases. Untreated group: normal care during four phases, with 0.9% saline gavage at Phase II and Phase III (the volume of saline was the same as the volume of lactose and bacteria solution used in the other groups). Model group: normal care at Phase I, followed by lactose solution gavage (12 mg of lactose per 20 g of body weight) during phases II and III, and 0.9% saline gavage with the same volume as that of the bacteria solution used in the other groups. Control group: normal care at Phase I, followed by lactose solution gavage (12 mg of lactose per 20 g of body weight) during phases II and III, and 0.3 mL of control bacteria solution gavage (BL21: *pETDuet1-0*, OD_600_ = 1) during Phase III. Test group: normal care at Phase I, followed by lactose solution gavage (12 mg of lactose per 20 g of body weight) during phases II and III, and 0.3 mL of engineered bacteria solution gavage (BL21: *pETDuet1-0*, OD_600_ = 1) during Phase III. All mice in the groups were raised under normal conditions during Phase IV. Three feces pellets were collected from each mouse at 2:00 PM each day.

### DNA extraction and 16S rRNA gene sequencing

The total genomic DNA from the feces samples was extracted using a QIAamp® Fast DNA Stool Mini Kit (QIAGEN) according to the manufacturer’s protocol. The DNA concentration was determined with a Qubit3.0 Fluorometer. A 20–30 ng quantity of DNA was used to generate amplicons. The V3 and V4 regions were amplified using forward primers containing the sequence “CCTACGGRRBGCASCAGKVRVGAAT” and reverse primers containing the sequence “GGACTACNVGGGTWTCTAATCC.” Simultaneously, indexed adapters were added to the ends of the 16S rDNA amplicons to generate indexed libraries ready for downstream NGS sequencing on Illumina MiSeq. PCR reactions were performed in triplicate 25 μL mixtures containing 2.5 μL of TransStart Buffer, 2 μL of dNTPs, 1 μL of each primer, and 20 ng of template DNA. The concentrations of the DNA libraries were validated by Qubit3.0 Fluorometer. After the DNA libraries were quantified at 10 nM, they were multiplexed and loaded on an Illumina MiSeq instrument according to the manufacturer’s instructions. Sequencing was performed using a PE250/300 paired-end approach. The sequencing data are accessible in NCBI SRA (with project accession number SRP152069).

### Sequence analysis

The raw multiplexed-paired-end sequences were firstly input to QIIME2 (version 2020.11.0) [36] and were demultiplexed using “qiime cutadapt demux-paired” with “--p-error-rate 0.” The primers of demultiplexed sequences were then trimmed using “qiime cutadapt trim-paired” with “--p-match-adapter-wildcards --p-match-read-wildcards --p-discard-untrimmed.” The trimmed sequences were quality-controlled using “qiime dada2 denoise-paired” with “--p-trunc-len-f 275 --p-trunc-len-r 220 --p-n-threads 20 --p-min-fold-parent-over-abundance 4.” The dada2-produced feature table was rarefied to 4000 reads per sample using “qiime feature-table rarefy” with “--p-sampling-depth 4000” based on the curve plateaus of alpha diversity. The dada2-produced representative sequences were taxonomically annotated using “qiime feature-classifier classify-sklearn” against the V3–V4 region of the Silva 138 database [37]. The taxonomic annotations were integrated into the rarefied feature table using “qiime taxa collapse.”

### Jensen-Shannon divergence distance

The Jensen-Shannon divergence (JSD) distance *D*(*a*, *b*) between samples *a* and *b* is defined as

1 $$ D\left(a,b\right)=\sqrt{JSD\left({p}_a,{p}_b\right)} $$

where *p*_*a*_ and *p*_*a*_ are the abundance distributions of samples *a* and *b* and *JSD*(*x*, *y*) is the JSD between two abundance distributions of genus *x* and genus *y* defined as

2 $$ JSD\left(x,y\right)=\frac{1}{2} KLD\left(x,m\right)+\frac{1}{2} KLD\left(y,m\right) $$

where $m=\frac{x+y}{2}$ and *KLD*(*x*, *y*) is the Kullback-Leibler divergence between *x* and *y* defined as

3 $$ KLD\left(x,y\right)=\sum \limits_i\ {x}_i\log \frac{x_i}{y_i} $$

We added a pseudocount of 0.000001 to the abundance distributions and renormalized them to avoid zero in the numerator and/or denominator of Eq. (3).

### Principal coordinate analysis

R function “dudi.pco” in the R package “ade4” was used to perform principal coordinate analysis (PCoA) on the JSD distance matrix. R package “ggplot2” was finally used to visualize the results.

### Microbiota network construction

Amplicon sequence variants (ASVs) with at least 20% prevalence among all samples were used to construct microbiota network. The unweighted-glasso network was constructed using “spiec.easi” in R package “SpiecEasi” (version 1.1.0) [38], with “method = ‘glasso’, lambda.min.ratio = 0.01, nlambda = 20, pulsar.params = list (rep.num = 50)”. Samples from the untreated group at days 7 and 11 were used to construct the network of “normal condition.” Samples from the model and control at days 5, 7, and 11, and test groups at days 5 and 7 were used for the network of “lactose feeding.” Samples from the test group at days 11 and 13 were used for the network of “treatment.”

### Statistical analysis

For categorical metadata, samples were pooled into bins (Phase I/Phase II..., Day 3/Day 5/Day 7…) and significant features were identified using Mann-Whitney-Wilcoxon Test with Benjamini and Hochberg correction of *P* values.

## Supplementary Information

**Additional file 1 **Table S1 to S9. Detailed data of results. **Table S1:** Detailed sequences of all the parts of our designed circuits; **Table S2:** Raw data for the fluorescence measurement of the protein & promoter; **Table S3:** Raw data for the fluorescence measurement of the protein & protein interaction of CIII & CII; **Table S4:** Parameters for tri-stable circuit modelling; **Table S5:** The variation of pH, β-galactosidase activity and l-lactate dehydrogenase activity in in vitro experiment to confirm the functionality of the circuit; **Table S6:** The variation of pH of the mice colon in the *in vivo* experiment to confirm the functionality of the circuit; **Table S7:** The variation of the β-galactosidase of the mice feces in the *in vivo* experiment to confirm the functionality of the circuit. **Table S8:** The experiment design and fecal sample collection of the 21-days *in vivo* experiment to detect the variation of mice gut microbiota; **Table S9:** The mean abundance of ASVs in the network and their taxonomic annotations.

**Additional file 2.** Supplementary results. The supplementary results of fluorescence detection, testing colonisation of the bacteria, testing the cage effects on gut microbiota.

**Additional file 3 **The plasmid profile of *pETDuet1-1*.

**Additional file 4 **The plasmid profile of *pETDuet1-0*.

**Additional file 5.** Table S10 to S15. Detailed parameters of the methods. **Table S10:** Regular PCR reaction system; **Table S11:** Double enzyme reaction system; **Table S12:** Ligation system; **Table S13:** In-Fusion system; **Table S14:** Protocol for M9 minimal medium preparation; **Table S15:** Instrument setting of the plate reader for fluorescence measurement.

## Data Availability

The datasets generated and analyzed during the current study are available in the short read archive (SRA) section of National Center for Biotechnology Information, under accession SRP152069 [39].
