# Supplementary material for: An engineered genetic circuit for lactose intolerance alleviation
Source: BMC Biol. 2021 Jul 5;19:137. doi: 10.1186/s12915-021-01070-9 (PMC8259030; doi:10.1186/s12915-021-01070-9)
Supplement: Supplementary file 2 — Additional file 2. Supplementary results. The supplementary results of fluorescence detection, testing colonisation of the bacteria, testing the cage effects on gut microbiota. [file 12915_2021_1070_MOESM2_ESM.docx]

# An Engineered Genetic Circuit for Lactose Intolerance Alleviation Coupled with Gut Microbiota Recovery

Mingyue Cheng^1,2^^†^, Zhangyu Cheng^1,2†^, Yiyan Yu^1,2^, Wangjie Liu^1,2^, Ruihao Li^1,2^, Zhenyi Guo^1,2^, Jiyue Qin^1,2^, Zhi Zeng^1,2^, Lin Di^1,2^, Yufeng Mo^1,2^, Chunxiu Pan^1,2^, Yuanhao Liang^1,2^, Jinman Li^4^, Yigang Tong^4,5^, Yunjun Yan^1,3*^, Yi Zhan^1,2,3*^, Kang Ning^1,3*^

**Supplementary Results**

**Tri-stable switch characterization**

***Protein & promoter interaction***

Three pairs of protein & promoter were characterized: *cII* & pRE, *cI* & pR, *cro* & pRM. Using appropriate primers, PCR was carried out to amplify *cII*, *cII*-TT, pRE-RBS, and *GFP*. *cII*-TT-pRE-RBS-*GFP* was constructed by In-Fusion cloning method, and pRE-RBS-*GFP* was successively constructed through enzyme digestion and ligation to serve as the control (Fig. S1a). Likewise, *cI*-TT-pR-RBS-*GFP* was constructed together with its control pR-RBS-*GFP* for the characterization of *cI*&pR (Fig. S1b). Moreover, *cro*-TT-pRM-RBS-*GFP* and pRM-RBS-*GFP* for the characterization of *cro* & pRM (Fig. S1c).

Then plasmids were transformed into *E.coli* BL21 for gene expression.

**
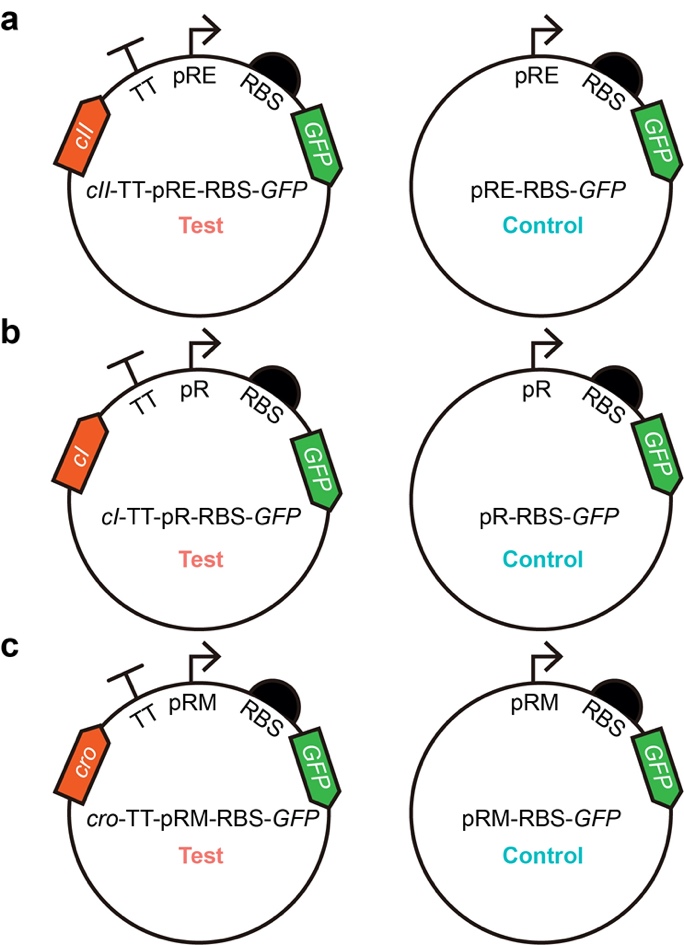
**

**Fig. S1** The diagram of plasmid construction for protein & promoter characterization. The genetic parts were arranged as plotted to construct the plasmid for the fluorescence detection.

GFP was utilized as a reporter to characterize the interaction between protein and promoter. The fluorescence measurement curve of *cII* & pRE showed that the test group was distinguished from its control group, presenting higher fluorescence intensity (Fig. S2a and d, Additional file 1: Table S2). It turned out that *cII*, as a transcription activator, can efficiently initiate the gene transcription after the pRE. As for interactions between *cI* & pR and *cro* & pRM, fluorescence intensity was decreased in the test groups, as compared to their corresponding control groups (Fig. S2b and e, Fig. S2c and f, Additional file 1: Table S2). It turned out that *cI* and *cro* can repress the gene transcription at the downstream of pR and pRM, respectively. Notably, the repression activity of *cro* was higher than that of *cI*.

**
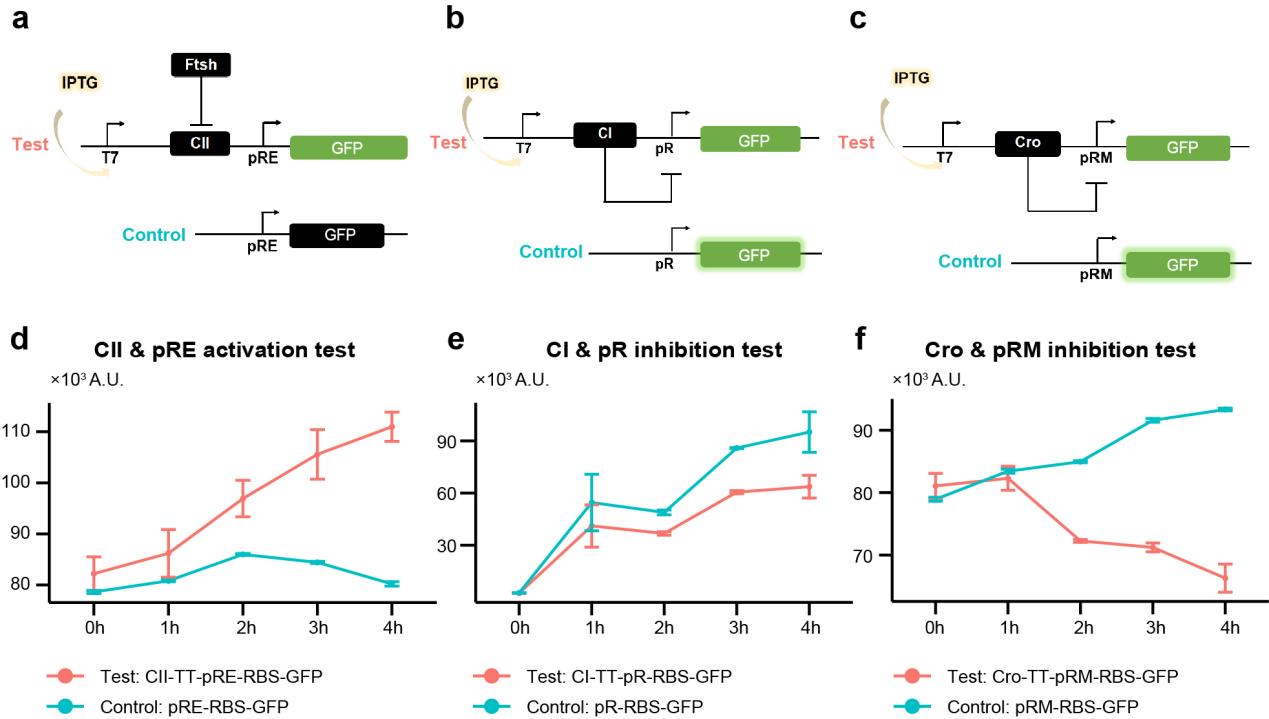
**

**Fig. S2** Theoretical experimental prototype and fluorescence detection of interactions between *cII* & pRE, *cI* & pR, and *cro* & pRM. The theoretical designs are plotted in **a**, **b**, and **c**, verified in **d**, **e**, and **f**, respectively.

***Protein & protein interaction***

To test whether *cIII* can protect *cII* from being degraded by Ftsh by competitive inhibition, the circuit construction for this purpose was based on the intermediate products of the *cII* & pRE test circuits above (Fig. S3). The fluorescence measurement curve of the test circuit increased more evidently than that of other two control circuits (Fig. S4, Additional file 1: Table S3), suggesting that tandem-expressed *cIII* can efficiently protect *cII* from being degraded by Ftsh expression.


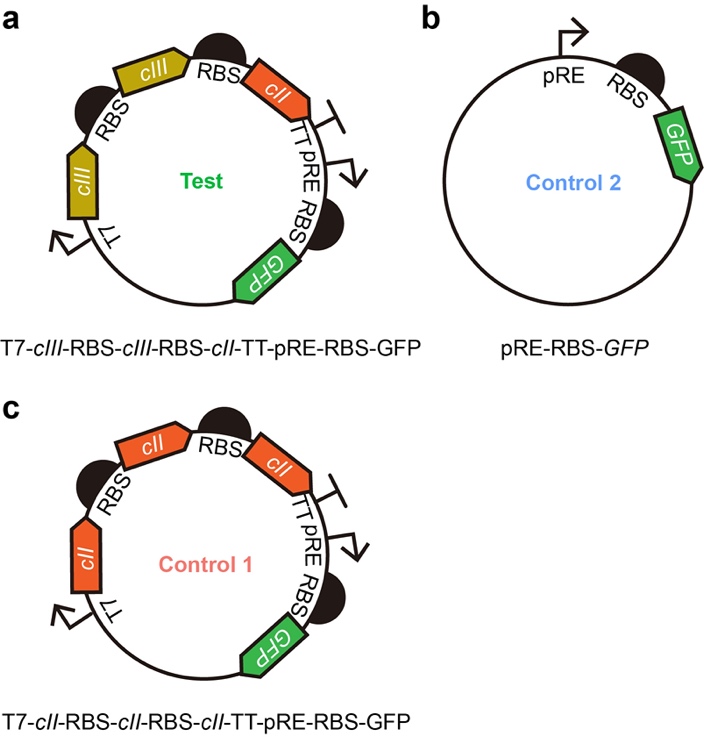


**Fig. S3** The diagram of plasmid construction for protein & protein characterization. The genetic parts were arranged as plotted to construct the plasmid for the fluorescence detection.


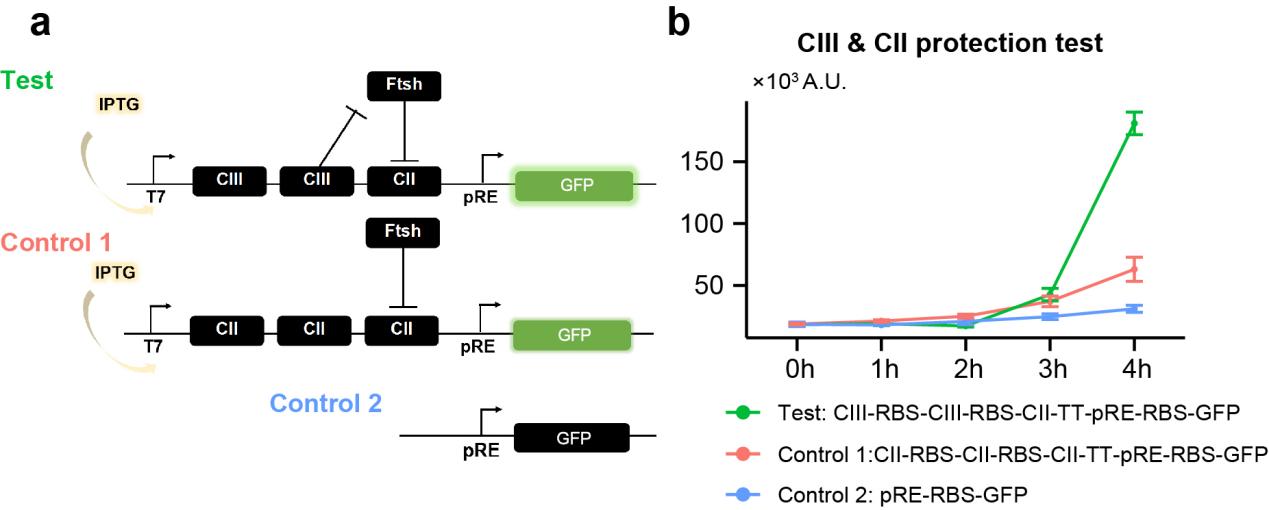


**Fig. S4** Ideal experimental prototype and fluorescence detection of interactions between *cIII* & *cII*. The theoretical designs are plotted in **a**, and verified in **b**.

**Tri-stable circuit modeling**

To simulate the reaction of this tri-stable pathway in engineering bacteria, we established a delay differential equation system based on Michaelis-Menten equation and chemical reaction kinetics (Detailed parameters are recorded in Additional file1: Table S4. Formulas are displayed below), and then translated the system into mathematical language.

$\frac{d[mCI]}{dt}={trc}_{pulse2}{copynum}_{theplasmid}-{deg}_{mCI}[mCI]$ (1)

$\frac{d[mCII]}{dt}={trc}_{pulse1}{copynum}_{theplasmid}+{copynum}_{theplasmid}\left( \frac{V_{maxmCII}\left[ CII\left( t-\tau_{2} \right) \right]}{K_{mCII}+\left[ CII\left( t-\tau_{2} \right) \right]} \right)-{deg}_{mCII}[mCII]$ (2)

$\frac{d[mCro]}{dt}={trc}_{pulse1}{copynum}_{theplasmid}+{copynum}_{theplasmid}\left( \frac{V_{maxmCro}\left[ CII\left( t-\tau_{3} \right) \right]}{K_{mCII}+\left[ CII\left( t-\tau_{3} \right) \right]} \right)-{deg}_{mCro}[mCro]$ (3)

$\frac{d[mCIII]}{dt}={trc}_{pulse1}{copynum}_{theplasmid}+{copynum}_{theplasmid}V_{maxmCIII}\left( 1-\frac{\left[ {CI}_{2}\left( t-\tau_{5} \right) \right]^{2}}{K_{m{CI}_{2}}+\left[ {CI}_{2}\left( t-\tau_{5} \right) \right]^{2}} \right)-{deg}_{mCro}[mCro]$ (4)

$\frac{d[CI]}{dt}={trl}_{CI}[mCI]-{deg}_{CI}[CI]-k_{1}{[CI]}^{2}+k_{2}[{CI}_{2}]$ (5)

$\frac{d[{CI}_{2}]}{dt}=k_{1}{[CI]}^{2}-k_{2}[{CI}_{2}]$ (6)

$\frac{d[mGene\_of\_interest\_1]}{dt}={copynum}_{theplasmid}V_{mGene\_of\_interest\_1}\left( 1-\frac{\left[ {CI}_{2}\left( t-\tau_{1} \right) \right]^{2}}{K_{m{CI}_{2}}+\left[ {CI}_{2}\left( t-\tau_{1} \right) \right]^{2}} \right)-{deg}_{mGene\_of\_interest\_1}[mGene\_of\_interest\_1$] (7)

$\frac{d[mGene\_of\_interest\_2]}{dt}={copynum}_{theplasmid}V_{mGene\_of\_interest\_2}\left( 1-\frac{\left[ Cro\left( t-\tau_{4} \right) \right]^{2}}{K_{mCro}+\left[ Cro\left( t-\tau_{4} \right) \right]^{2}} \right)-{deg}_{mGene\_of\_interest\_2}[mGene\_of\_interest\_2]$ (8)

$\frac{d[CII]}{dt}={trl}_{CII}[mCII]-{deg}_{CII}[CII]-\frac{V_{CIImax}[CII]}{\left( 1+\frac{[CIII]}{K_{mCIII}} \right)K_{mCII}+[CII]}$ (9)

$\frac{d[Cro]}{dt}={trl}_{mCro}[mCro]-{deg}_{Cro}[Cro$] (10)

$\frac{d[CIII]}{dt}={trl}_{CIII}[mCIII]-{deg}_{CIII}[CIII]-\frac{V_{CIIImax}[CII]}{\left( 1+\frac{[CII]}{K_{mCII}} \right)K_{mCIII}+[CIII]}$ (11)

$\frac{d[Gene\_of\_interest\_1]}{dt}={trl}_{Gene\_of\_interest\_1}[Gene\_of\_interest\_1]-{deg}_{Gene\_of\_interest\_1}[Gene\_of\_interest\_1]$ (12)

$\frac{d[Gene\_of\_interest\_2]}{dt}={trl}_{Gene\_of\_interest\_2}[Gene\_of\_interest\_2]-{deg}_{Gene\_of\_interest\_2}[Gene\_of\_interest\_2]$ (13)

## *Test of switch function*

To determine whether the designed tri-stable circuit can work properly, and to understand how the signal leads to the switch of the pathway, we adjust the strength ratio of pulse1 input to pulse2 input to observe the state switch of the pathway.

Our switching circuit faithfully fulfilled its duty to express only one protein under a specific signal input. While the other one is suppressed to be at a low level of expression. Besides, when the ratio of the two signals arrives at 10, the switching is enabled (Fig. S5).


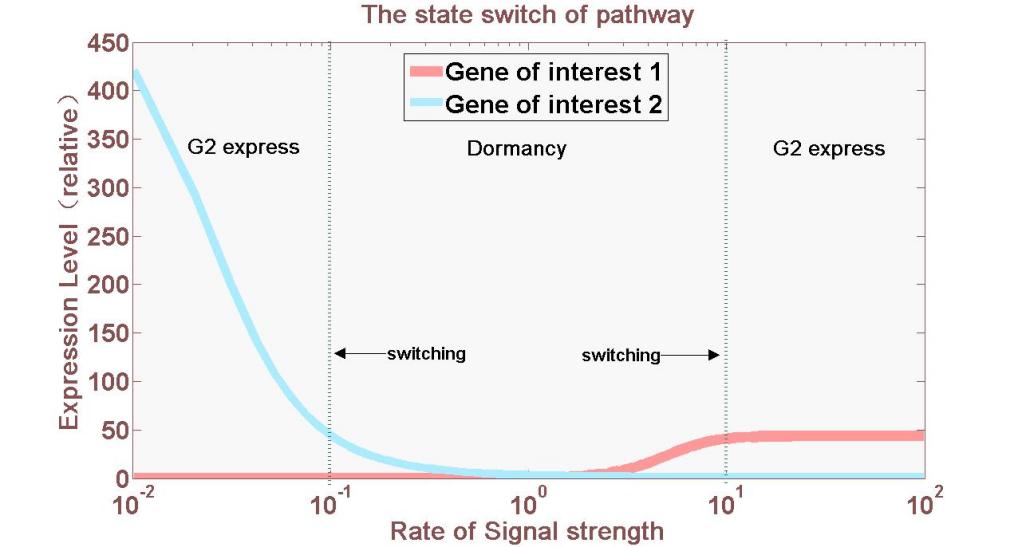


**Fig. S5** Modeling results on relative protein expression level versus rate of signal strength.

***In vivo* mice trial**

## *Colonization of the* BL21: *pETDuet1-1*

In the *in vivo* experiment for pH variation of the mice colon, we have collected the colon content and the faeces of the mice at each time point after one-week administration of the BL21: *pETDuet1-1*. Besides measuring pH values, we also used the spread plate method to obtain a single colony for either colon content or faecal samples. We performed PCR on them to detect whether the BL21: *pETDuet1-1* have colonized in the mice colon, using the forward primer 5' AATCATAGCCTGAGCGACGG 3' and the reverse primer 5' CTGTGATCGTTGCGCTGATG 3'. To verify the successful long-term colonization of the bacteria, we set an additional group of four mice administrated with the BL21: *pETDuet1-1* the same as that of the Test group for one week and collected their faeces at the time point of 8h and 24h.

Therefore, the examined samples include the used colon content and the faecal samples of the Test group at the time point of 2h, 3h, 4h, 6h (Fig. S6a), and the faecal samples of the additional group at the time point of 8h and 24h (Fig. S6b). Each of the mice has been confirmed to be colonized by the BL21*: pETDuet1-1*. Additionally, the colonization can last at least 24 hrs.


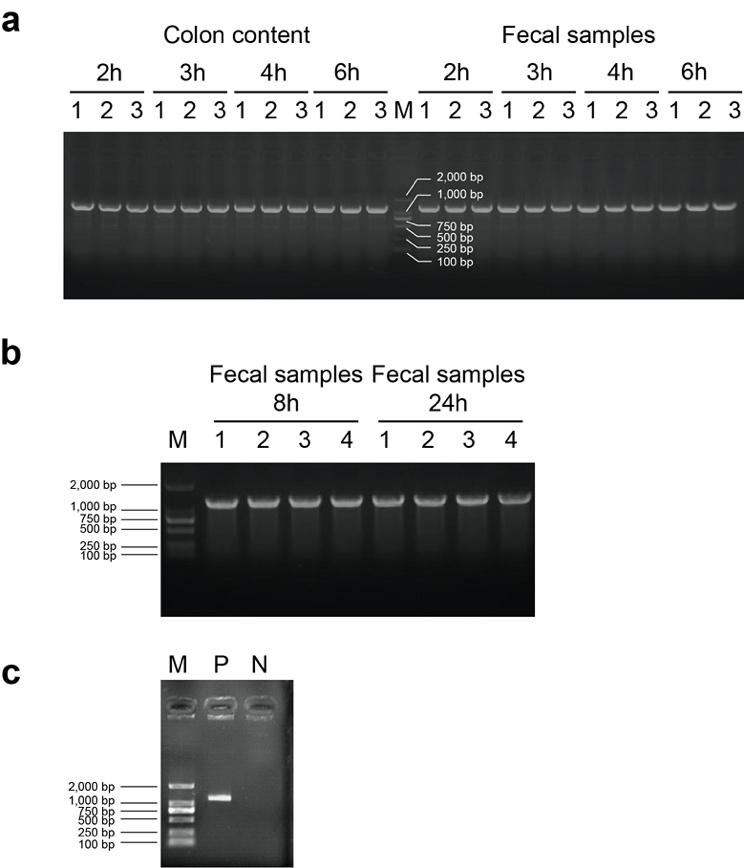


**Fig. S6** PCR results to confirm the colonization of the BL21: *pETDuet1-1*. **a** The PCR results using samples of the Test group with random selection of three replicates out of four. **b** The PCR results using samples of the additional mice group administrated with the BL21: *pETDuet1-1*. **c** The PCR results of the positive control (P) and the negative control (N).

## *Weight variation and intestinal anatomy*

In the 21-day murine experiment, the variations in normalized mouse weights of the four groups including the Untreated group, the Model group, the Control group, and the Test group are shown in Fig. S7. The Model and Control groups, which received pulse lactose feeding, showed a downward trend in their weights from days 3 to 9 and days 9 to 15, while the Untreated and Test groups showed a downward trend in their weights from days 3 to 9 but then recovered from days 9 to 15. The variation of weight can be related to many factors, such as the bacteria inoculation, lactose gavage and the gut microbiota. The similar variation patterns in the weights of the Untreated and Test groups suggested that the administration of the engineered bacteria might assist in the recovery of the mouse weight. However, theses results of the weight variation were not statistically significant (P > 0.05, Wilcox test). It might be due to a limitation of the experimental design that the dose of the lactose used in this study was unable to cause a quantifiable effect on weight of mice, and other measurable symptoms of LI.


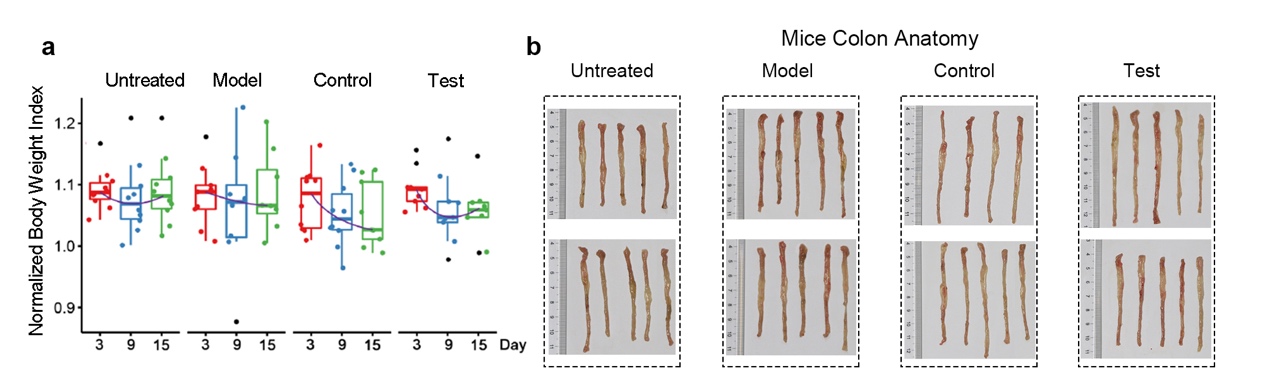
**Fig. S7** The variation of mice weight and mice intestinal anatomy. **a** The mouse body weights were normalized according to the first day’s value. The normalized body weight index for each mouse at days 3, 9, and 15 was shown in box plot with trend-line. **b** The intestinal tissue anatomy of every mouse from all of the four groups is exhibited, except for that of mice who died during the experiment.

## *No evident cage effect observed*

To test whether there were cage effects on Fig. 4 (main text) in this study, we plotted the variation of mice's gut microbiota in each cage (Fig. S8). Some slight differences in gut microbiota were observed in a single day, while the overall trend remained similar. Additionally, no matter in which cage, the Test group's gut microbiota was still observed to recover at the same level as that in the Untreated group. In contrast, the other two groups were still observed to be inhibited. Moreover, when comparing all samples of each pair of the cages, no significant difference was observed (Wilcox test). Therefore, there was no evident cage effect on the conclusion of this study.

**
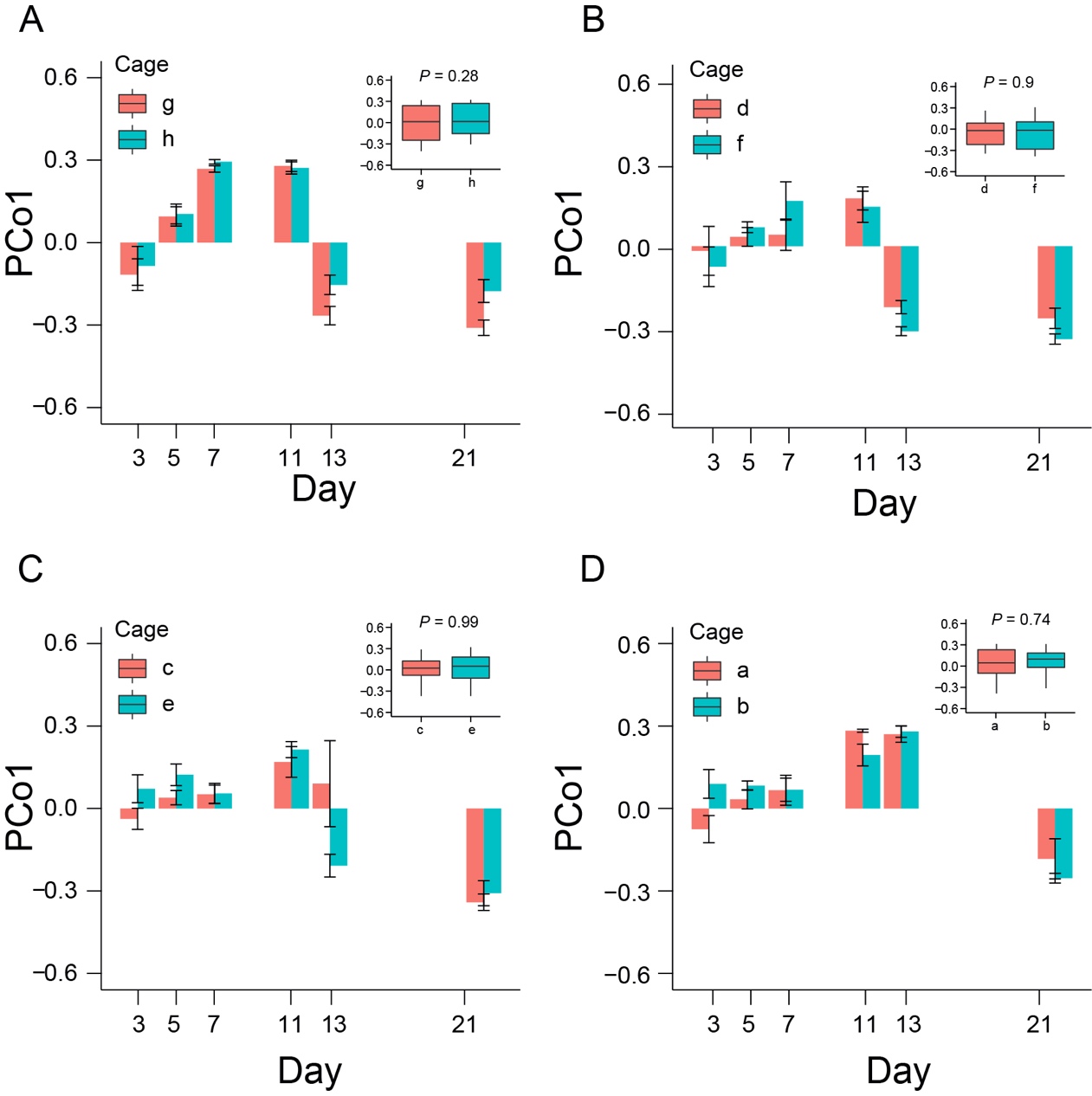
**

**Fig. S8** Comparing gut microbiota compositions between cages. The mean ± s.e.m. of PCo1 coordinates of JSD PCoA plot (Fig. 4 in the main text) from eight cages of the four trial groups across 21 days. *P < 0.05, **P < 0.01, ***P < 0.001; ns, not significant. Wilcox test.
