# Supplementary material for: An engineered genetic circuit for lactose intolerance alleviation
Source: BMC Biol. 2021 Jul 5;19:137. doi: 10.1186/s12915-021-01070-9 (PMC8259030; doi:10.1186/s12915-021-01070-9)
Supplement: Supplementary file 3 — Additional file 3 The plasmid profile of pETDuet1-1. [file 12915_2021_1070_MOESM3_ESM.pdf]

Created by GenSmart Design, GenScript

Created time: 08:49:53, 03/26/2020

1. Map

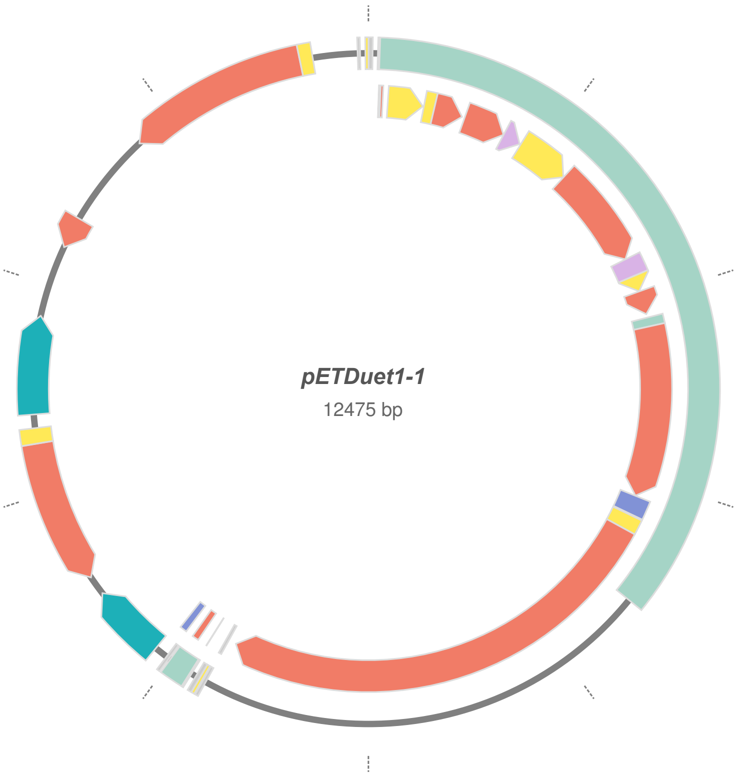

2. Construct Information

Construct name	*pETDuet1-1*
Construct size (bp)	12475 bp

3. Sequence

GGAATTGTGAGCGGATAACAATTCCCCTCTAGAAATAATTTTGTTTAACTTTAAGAAGGAGATATACCATGGGCAGCAGCCATCACCATCATCACCACAGCCAGGATCCGAATTCGAGCT
CCTTAACACTCGCCTATTGTTAAGGGGAGATCTTTATTAAAACAAATTGAAATTCTTCCTCTATATGGTACCCGTCGTCGGTAGTGGTAGTAGTGGTGTCGGTCCTAGGCTTAAGCTCGA

lac operator RBS MCS-1 M

H H H H H H

6xHis

10 20 30 40 50 60 70 80 90 100 110 120

CGGCGCGCCTGCAGTCTCACTGGTGAAAAGAAAAACCACCCTGGCGCCCAATACGCAAACCGCCTCTCCCCGCGCGTTGGCCGATTCATTAATGCAGCTGGCACGACAGGTTTCCCGACT
GCCGCGCGGACGTCAGAGTGACCACTTTTCTTTTTGGTGGGACCGCGGGTTATGCGTTTGGCGGAGAGGGGCGCGCAACCGGCTAAGTAATTACGTCGACCGTGCTGTCCAAAGGGCTGA

plac-m

130 140 150 160 170 180 190 200 210 220 230 240

GGAAAGCGGGCAGTGAGCGCAACGCAATTCCACGCTGTATCCGATGTCAGTAGGCACCCCAGGCTTTACACTTTATGCTTCCGGCTCGTATGTTGTGTGGAATTGTGAGCGGATAACAAT
CCTTTCGCCCGTCACTCGCGTTGCGTTAAGGTGCGACATAGGCTACAGTCATCCGTGGGGTCCGAAATGTGAAATACGAAGGCCGAGCATACAACACACCTTAACACTCGCCTATTGTTA

250 260 270 280 290 300 310 320 330 340 350 360

TTCACACAGGAAACAGCTTACACGAGAGCCTCGTTGCGTTTGTTTGCACGAACCATATGTAAGTATTTCCTTAGATATTAAAGAGGAGAAATACACGATGAAAGCGTATTGGGACTCTTT
AAGTGTGTCCTTTGTCGAATGTGCTCTCGGAGCAACGCAAACAAACGTGCTTGGTATACATTCATAAAGGAATCTATAATTTCTCCTCTTTATGTGCTACTTTCGCATAACCCTGAGAAA

pRE M K A Y W D S L

cro

370 380 390 400 410 420 430 440 450 460 470 480

AACCAAAGAACAGCAGGGCGAGTTGGCCGGAAAAGTTGGCTCAACACCTGGCTACTTACGGCTGGTTTTCAATGGCTATAAAAAAGCCAGTTTTGTGCTGGCTAAAAAACTTGAGCAATA
TTGGTTTCTTGTCGTCCCGCTCAACCGGCCTTTTCAACCGAGTTGTGGACCGATGAATGCCGACCAAAAGTTACCGATATTTTTTCGGTCAAAACACGACCGATTTTTTGAACTCGTTAT
T K E Q Q G E L A G K V G S T P G Y L R L V F N G Y K K A S F V L A K K L E Q Y

490 500 510 520 530 540 550 560 570 580 590 600

CACATCAGGTGCAATTACGAAATCTGACTTAAGACCGGATATCTATCCGAAAGATTAGAAAGAGGAGAAATACACGATGGTTCGTGCAAACAAACGCAACGAGGCTCTACGAATCGAGAG
GTGTAGTCCACGTTAATGCTTTAGACTGAATTCTGGCCTATAGATAGGCTTTCTAATCTTTCTCCTCTTTATGTGCTACCAAGCACGTTTGTTTGCGTTGCTCCGAGATGCTTAGCTCTC
T S G A I T K S D L R P D I Y P K D *

M V R A N K R N E A L R I E S

cII

610 620 630 640 650 660 670 680 690 700 710 720

TGCGTTGCTTAACAAAATCGCAATGCTTGGAACTGAGAAGACAGCGGAAGCTGTGGGCGTTGATAAGTCGCAGATCAGCAGGTGGAAGAGGGACTGGATTCCAAAGTTCTCAATGCTGCT
ACGCAACGAATTGTTTTAGCGTTACGAACCTTGACTCTTCTGTCGCCTTCGACACCCGCAACTATTCAGCGTCTAGTCGTCCACCTTCTCCCTGACCTAAGGTTTCAAGAGTTACGACGA
A L L N K I A M L G T E K T A E A V G V D K S Q I S R W K R D W I P K F S M L L

730 740 750 760 770 780 790 800 810 820 830 840

TGCTGTTCTTGAATGGGGGGTCGTTGACGACGACATGGCTCGATTGGCGCGACAAGTTGCTGCGATTCTCACCAATAAAAAACGCCCGGCGGCAACCGAGCGTTCTGAACAAATCCAGAT
ACGACAAGAACTTACCCCCCAGCAACTGCTGCTGTACCGAGCTAACCGCGCTGTTCAACGACGCTAAGAGTGGTTATTTTTTGCGGGCCGCCGTTGGCTCGCAAGACTTGTTTAGGTCTA
A V L E W G V V D D D M A R L A R Q V A A I L T N K K R P A A T E R S E Q I Q M

850 860 870 880 890 900 910 920 930 940 950 960

GGAGTTCTGACCAGGCATCAAATAAAACGAAAGGCTCAGTCGAAAGACTGGGCCTTTCGTTTTATCTGTTGTTTGTCGGTGAACGCTCTCTACTAGAGTCACACTGGCTCACCTTCGGGT
CCTCAAGACTGGTCCGTAGTTTATTTTGCTTTCCGAGTCAGCTTTCTGACCCGGAAAGCAAAATAGACAACAAACAGCCACTTGCGAGAGATGATCTCAGTGTGACCGAGTGGAAGCCCA
E F *
TT
10 20 30 40 50 60 70 80 90 100 110 120

GGGCCTTTCTGCGTTTATATACTAGAGTGTCCGTGCTGGGAAACTGGCGGGGAACTTTTAGAGATAACCCTCCGAATTGCTGGCAAATTTCTGATGAAATTTCTCCGCGAAGCCCACATG
CCCGGAAAGACGCAAATATATGATCTCACAGGCACGACCCTTTGACCGCCCCTTGAAAATCTCTATTGGGAGGCTTAACGACCGTTTAAAGACTACTTTAAAGAGGCGCTTCGGGTGTAC
patp2
130 140 150 160 170 180 190 200 210 220 230 240

AACTACCCCCGTTTACCCTCAAAATAAGCCCTGTGACACACATAACACCCCCTAATCGTACCCGCTCACACGCTATTTTCGAGGTGTGGTTCGCCTTCGGAAACGAATGCCCCCGCCCCA
TTGATGGGGGCAAATGGGAGTTTTATTCGGGACACTGTGTGTATTGTGGGGGATTAGCATGGGCGAGTGTGCGATAAAAGCTCCACACCAAGCGGAAGCCTTTGCTTACGGGGGCGGGGT
250 260 270 280 290 300 310 320 330 340 350 360

CTTGGATAAAAGACGTTAACACCTGTTAGTCTATAACGCGGGTTGAACCGAGAAACCCCTCAAGGCAGCAGACAATAGCCGCAAGGGGTTTTGCGGAGCACGTCCCCTGTGATCGTTGCG
GAACCTATTTTCTGCAATTGTGGACAATCAGATATTGCGCCCAACTTGGCTCTTTGGGGAGTTCCGTCGTCTGTTATCGGCGTTCCCCAAAACGCCTCGTGCAGGGGACACTAGCAACGC
370 380 390 400 410 420 430 440 450 460 470 480

CTGATGTGCGACGGAGTCCGAAAGAGGAGAAATACTAGATGAGCACAAAAAAGAAACCATTAACACAAGAGCAGCTTGAGGACGCACGTCGCCTTAAAGCAATTTATGAAAAAAAGAAAA
GACTACACGCTGCCTCAGGCTTTCTCCTCTTTATGATCTACTCGTGTTTTTTCTTTGGTAATTGTGTTCTCGTCGAACTCCTGCGTGCAGCGGAATTTCGTTAAATACTTTTTTTCTTTT
M S T K K K P L T Q E Q L E D A R R L K A I Y E K K K
cI
490 500 510 520 530 540 550 560 570 580 590 600

ATGAACTTGGCTTATCCCAGGAATCTGTCGCAGACAAGATGGGGATGGGGCAGTCAGGCGTTGGTGCTTTATTTAATGGCATCAATGCATTAAATGCTTATAACGCCGCATTGCTTGCAA
TACTTGAACCGAATAGGGTCCTTAGACAGCGTCTGTTCTACCCCTACCCCGTCAGTCCGCAACCACGAAATAAATTACCGTAGTTACGTAATTTACGAATATTGCGGCGTAACGAACGTT
N E L G L S Q E S V A D K M G M G Q S G V G A L F N G I N A L N A Y N A A L L A
610 620 630 640 650 660 670 680 690 700 710 720

AAATTCTCAAAGTTAGCGTTGAAGAATTTAGCCCTTCAATCGCCAGAGAAATCTACGAGATGTATGAAGCGGTTAGTATGCAGCCGTCACTTAGAAGTGAGTATGAGTACCCTGTTTTTT
TTTAAGAGTTTCAATCGCAACTTCTTAAATCGGGAAGTTAGCGGTCTCTTTAGATGCTCTACATACTTCGCCAATCATACGTCGGCAGTGAATCTTCACTCATACTCATGGGACAAAAAA
K I L K V S V E E F S P S I A R E I Y E M Y E A V S M Q P S L R S E Y E Y P V F
730 740 750 760 770 780 790 800 810 820 830 840

CTCATGTTCAGGCAGGGATGTTCTCACCTGAGCTTAGAACCTTTACCAAAGGTGATGCGGAGAGATGGGTAAGCACAACCAAAAAAGCCAGTGATTCTGCATTCTGGCTTGAGGTTGAAG
GAGTACAAGTCCGTCCCTACAAGAGTGGACTCGAATCTTGGAAATGGTTTCCACTACGCCTCTCTACCCATTCGTGTTGGTTTTTTCGGTCACTAAGACGTAAGACCGAACTCCAACTTC
S H V Q A G M F S P E L R T F T K G D A E R W V S T T K K A S D S A F W L E V E
850 860 870 880 890 900 910 920 930 940 950 960

GenScript
Make Research Easy

GTAATTCCATGACCGCACCAACAGGCTCCAAGCCAAGCTTTCCTGACGGAATGTTAATTCTCGTTGACCCTGAGCAGGCTGTTGAGCCAGGTGATTTCTGCATAGCCAGACTTGGGGGTG
CATTAAGGTACTGGCGTGGTTGTCCGAGGTTCGGTTCGAAAGGACTGCCTTACAATTAAGAGCAACTGGGACTCGTCCGACAACTCGGTCCACTAAAGACGTATCGGTCTGAACCCCCAC
G N S M T A P T G S K P S F P D G M L I L V D P E Q A V E P G D F C I A R L G G

10 20 30 40 50 60 70 80 90 100 110 120

ATGAGTTTACCTTCAAGAAACTGATCAGGGATAGCGGTCAGGTGTTTTTACAACCACTAAACCCACAGTACCCAATGATCCCATGCAATGAGAGTTGTTCCGTTGTGGGGAAAGTTATCG
TACTCAAATGGAAGTTCTTTGACTAGTCCCTATCGCCAGTCCACAAAAATGTTGGTGATTTGGGTGTCATGGGTTACTAGGGTACGTTACTCTCAACAAGGCAACACCCCTTTCAATAGC
D E F T F K K L I R D S G Q V F L Q P L N P Q Y P M I P C N E S C S V V G K V I

130 140 150 160 170 180 190 200 210 220 230 240

CTAGTCAGTGGCCTGAAGAGACGTTTGGCTGACCAGGCATCAAATAAAACGAAAGGCTCAGTCGAAAGACTGGGCCTTTCGTTTTATCTGTTGTTTGTCGGTGAACGCTCTCTACTAGAG
GATCAGTCACCGGACTTCTCTGCAAACCGACTGGTCCGTAGTTTATTTTGCTTTCCGAGTCAGCTTTCTGACCCGGAAAGCAAAATAGACAACAAACAGCCACTTGCGAGAGATGATCTC
A S Q W P E E T F G *

TT

250 260 270 280 290 300 310 320 330 340 350 360

TCACACTGGCTCACCTTCGGGTGGGCCTTTCTGCGTTTATAAAATCTATCACCGCAAGGGATAAATATCTAACACCGTGCGTGTTGACTATTTTACCTCTGGCGGTGATAATGGTTGCAT
AGTGTGACCGAGTGGAAGCCCACCCGGAAAGACGCAAATATTTTAGATAGTGGCGTTCCCTATTTATAGATTGTGGCACGCACAACTGATAAAATGGAGACCGCCACTATTACCAACGTA

pR

370 380 390 400 410 420 430 440 450 460 470 480

GTACTAAGGAGGTTGAAAGAGGAGAAATACACGATGCAATATGCCATTGCAGGGTGGCCTGTTGCTGGCTGCCCTTCCGAATCTTTACTTGAACGAATCACCCGTAAATTACGTGACGGA
CATGATTCCTCCAACTTTCTCCTCTTTATGTGCTACGTTATACGGTAACGTCCCACCGGACAACGACCGACGGGAAGGCTTAGAAATGAACTTGCTTAGTGGGCATTTAATGCACTGCCT
M Q Y A I A G W P V A G C P S E S L L E R I T R K L R D G

cIII

490 500 510 520 530 540 550 560 570 580 590 600

TGGAAACGCCTTATCGACATACTTAATCAGCCAGGAGTCCCAAAGAATGGATCAAACACTTATGGCTATCCAGACTAATACTAGAAAGAGGAGAAATACACGATGAAAAAGACAGCTATC
ACCTTTGCGGAATAGCTGTATGAATTAGTCGGTCCTCAGGGTTTCTTACCTAGTTTGTGAATACCGATAGGTCTGATTATGATCTTTCTCCTCTTTATGTGCTACTTTTTCTGTCGATAG
W K R L I D I L N Q P G V P K N G S N T Y G Y P D *

ompA

610 620 630 640 650 660 670 680 690 700 710 720

GCGATTGCAGTGGCACTGGCAGGTTTCGCTACCGTCGCTCAGGCTATGATTATTTCCGCAGCCAGCGATTATCGCGCCGCAGCGCAACGCATTCTGCCGCCGTTCCTGTTCCACTATATG
CGCTAACGTCACCGTGACCGTCCAAAGCGATGGCAGCGAGTCCGATACTAATAAAGGCGTCGGTCGCTAATAGCGCGGCGTCGCGTTGCGTAAGACGGCGGCAAGGACAAGGTGATATAC
M I I S A A S D Y R A A A Q R I L P P F L F H Y M

lldD

730 740 750 760 770 780 790 800 810 820 830 840

GATGGTGGTGCATATTCTGAATACACGCTGCGCCGCAACGTGGAAGATTTGTCAGAAGTGGCGCTGCGCCAGCGTATTCTGAAAAACATGTCCGACTTAAGCCTGGAAACGACGCTGTTT
CTACCACCACGTATAAGACTTATGTGCGACGCGGCGTTGCACCTTCTAAACAGTCTTCACCGCGACGCGGTCGCATAAGACTTTTTGTACAGGCTGAATTCGGACCTTTGCTGCGACAAA
D G G A Y S E Y T L R R N V E D L S E V A L R Q R I L K N M S D L S L E T T L F

850 860 870 880 890 900 910 920 930 940 950 960

GenScript USA, Inc.
860 Centennial Ave. Piscataway, NJ 08854
Tel: 1-732-885-9188

AATGAGAAATTGTCGATGCCGGTGGCACTGGCTCCGGTGGGTTTGTGTGGCATGTATGCGCGTCGTGGCGAAGTTCAGGCAGCCAAAGCGGCGGACGCGCATGGTATTCCGTTTACTCTC
TTACTCTTTAACAGCTACGGCCACCGTGACCGAGGCCACCCAAACACACCGTACATACGCGCAGCACCGCTTCAAGTCCGTCGGTTTCGCCGCCTGCGCGTACCATAAGGCAAATGAGAG
N E K L S M P V A L A P V G L C G M Y A R R G E V Q A A K A A D A H G I P F T L

10 20 30 40 50 60 70 80 90 100 110 120

TCGACGGTTTCCGTTTGCCCGATTGAAGAAGTCGCGCCAGCCATCAAGCGCCCAATGTGGTTCCAGCTTTATGTACTGCGCGATCGCGGCTTTATGCGTAACGCGCTGGAGCGAGCAAAA
AGCTGCCAAAGGCAAACGGGCTAACTTCTTCAGCGCGGTCGGTAGTTCGCGGGTTACACCAAGGTCGAAATACATGACGCGCTAGCGCCGAAATACGCATTGCGCGACCTCGCTCGTTTT
S T V S V C P I E E V A P A I K R P M W F Q L Y V L R D R G F M R N A L E R A K

130 140 150 160 170 180 190 200 210 220 230 240

GCAGCGGGTTGTTCGACGCTGGTTTTCACCGTGGATATGCCGACACCGGGCGCACGCTACCGTGATGCGCATTCAGGTATGAGCGGCCCGAACGCGGCAATGCGCCGCTACTTGCAAGCG
CGTCGCCCAACAAGCTGCGACCAAAAGTGGCACCTATACGGCTGTGGCCCGCGTGCGATGGCACTACGCGTAAGTCCATACTCGCCGGGCTTGCGCCGTTACGCGGCGATGAACGTTCGC
A A G C S T L V F T V D M P T P G A R Y R D A H S G M S G P N A A M R R Y L Q A

250 260 270 280 290 300 310 320 330 340 350 360

GTGACACATCCGCAATGGGCGTGGGATGTGGGCCTGAACGGTCGTCCACATGATTTAGGTAATATCTCAGCTTATCTCGGCAAACCGACCGGACTGGAAGATTACATCGGCTGGCTGGGG
CACTGTGTAGGCGTTACCCGCACCCTACACCCGGACTTGCCAGCAGGTGTACTAAATCCATTATAGAGTCGAATAGAGCCGTTTGGCTGGCCTGACCTTCTAATGTAGCCGACCGACCCC
V T H P Q W A W D V G L N G R P H D L G N I S A Y L G K P T G L E D Y I G W L G

370 380 390 400 410 420 430 440 450 460 470 480

AATAACTTCGATCCGTCCATCTCATGGAAAGACCTTGAATGGATCCGCGATTTCTGGGATGGCCCGATGGTGATCAAAGGGATCCTCGATCCGGAAGATGCGCGCGATGCAGTACGTTTT
TTATTGAAGCTAGGCAGGTAGAGTACCTTTCTGGAACTTACCTAGGCGCTAAAGACCCTACCGGGCTACCACTAGTTTCCCTAGGAGCTAGGCCTTCTACGCGCGCTACGTCATGCAAAA
N N F D P S I S W K D L E W I R D F W D G P M V I K G I L D P E D A R D A V R F

490 500 510 520 530 540 550 560 570 580 590 600

GGTGCTGATGGAATTGTGGTTTCTAACCACGGTGGCCGCCAGCTGGACGGTGTACTCTCTTCCGCCCGTGCACTGCCTGCTATTGCAGATGCGGTGAAAGGTGATATAGCCATTCTGGCG
CCACGACTACCTTAACACCAAAGATTGGTGCCACCGGCGGTCGACCTGCCACATGAGAGAAGGCGGGCACGTGACGGACGATAACGTCTACGCCACTTTCCACTATATCGGTAAGACCGC
G A D G I V V S N H G G R Q L D G V L S S A R A L P A I A D A V K G D I A I L A

610 620 630 640 650 660 670 680 690 700 710 720

GATAGCGGTATTCGTAACGGGCTTGATGTCGTGCGTATGATTGCGCTCGGTGCCGACACCGTACTGCTGGGTCGTGCTTTCTTGTATGCGCTGGCAACAGCGGGCCAGGCGGGTGTAGCT
CTATCGCCATAAGCATTGCCCGAACTACAGCACGCATACTAACGCGAGCCACGGCTGTGGCATGACGACCCAGCACGAAAGAACATACGCGACCGTTGTCGCCCGGTCCGCCCACATCGA
D S G I R N G L D V V R M I A L G A D T V L L G R A F L Y A L A T A G Q A G V A

730 740 750 760 770 780 790 800 810 820 830 840

AACCTGCTAAATCTGATCGAAAAAGAGATGAAAGTGGCGATGACGCTGACTGGCGCGAAATCGATCAGCGAAATTACGCAAGATTCGCTGGTGCAGGGGCTGGGTAAAGAGTTGCCTGCG
TTGGACGATTTAGACTAGCTTTTTCTCTACTTTCACCGCTACTGCGACTGACCGCGCTTTAGCTAGTCGCTTTAATGCGTTCTAAGCGACCACGTCCCCGACCCATTTCTCAACGGACGC
N L L N L I E K E M K V A M T L T G A K S I S E I T Q D S L V Q G L G K E L P A

850 860 870 880 890 900 910 920 930 940 950 960

GCACTGGCTCCCATGGCGAAAGGGAATGCGGCATAGCCAGGCATCAAATAAAACGAAAGGCTCAGTCGAAAGACTGGGCCTTTCGTTTTATCTGTTGTTTGTCGGTGAACGCTCTCTACT
CGTGACCGAGGGTACCGCTTTCCCTTACGCCGTATCGGTCCGTAGTTTATTTTGCTTTCCGAGTCAGCTTTCTGACCCGGAAAGCAAAATAGACAACAAACAGCCACTTGCGAGAGATGA
A L A P M A K G N A A *

TT

10 20 30 40 50 60 70 80 90 100 110 120

AGAGTCACACTGGCTCACCTTCGGGTGGGCCTTTCTGCGTTTATATACTAGTACGTTAAATCTATCACCGCAAGGGATAAATATCTAACACCGTGCGTGTTGACTATTTTACCTCTGGCG
TCTCAGTGTGACCGAGTGGAAGCCCACCCGGAAAGACGCAAATATATGATCATGCAATTTAGATAGTGGCGTTCCCTATTTATAGATTGTGGCACGCACAACTGATAAAATGGAGACCGC

pRM

130 140 150 160 170 180 190 200 210 220 230 240

GTGATAATGGTTGCAAAAGAGGAGAAATACACGATGACCATGATTACGGATTCACTGGCCGTCGTTTTACAACGTCGTGACTGGGAAAACCCTGGCGTTACCCAACTTAATCGCCTTGCA
CACTATTACCAACGTTTTCTCCTCTTTATGTGCTACTGGTACTAATGCCTAAGTGACCGGCAGCAAAATGTTGCAGCACTGACCCTTTTGGGACCGCAATGGGTTGAATTAGCGGAACGT
M T M I T D S L A V V L Q R R D W E N P G V T Q L N R L A

lacZ

250 260 270 280 290 300 310 320 330 340 350 360

GCACATCCCCCTTTCGCCAGCTGGCGTAATAGCGAAGAGGCCCGCACCGATCGCCCTTCCCAACAGTTGCGCAGCCTGAATGGCGAATGGCGCTTTGCCTGGTTTCCGGCACCAGAAGCG
CGTGTAGGGGGAAAGCGGTCGACCGCATTATCGCTTCTCCGGGCGTGGCTAGCGGGAAGGGTTGTCAACGCGTCGGACTTACCGCTTACCGCGAAACGGACCAAAGGCCGTGGTCTTCGC
A H P P F A S W R N S E E A R T D R P S Q Q L R S L N G E W R F A W F P A P E A

370 380 390 400 410 420 430 440 450 460 470 480

GTGCCGGAAAGCTGGCTGGAGTGCGATCTTCCTGAGGCCGATACTGTCGTCGTCCCCTCAAACTGGCAGATGCACGGTTACGATGCGCCCATCTACACCAACGTGACCTATCCCATTACG
CACGGCCTTTCGACCGACCTCACGCTAGAAGGACTCCGGCTATGACAGCAGCAGGGGAGTTTGACCGTCTACGTGCCAATGCTACGCGGGTAGATGTGGTTGCACTGGATAGGGTAATGC
V P E S W L E C D L P E A D T V V V P S N W Q M H G Y D A P I Y T N V T Y P I T

490 500 510 520 530 540 550 560 570 580 590 600

GTCAATCCGCCGTTTGTTCCCACGGAGAATCCGACGGGTTGTTACTCGCTCACATTTAATGTTGATGAAAGCTGGCTACAGGAAGGCCAGACGCGAATTATTTTTGATGGCGTTAACTCG
CAGTTAGGCGGCAAACAAGGGTGCCTCTTAGGCTGCCCAACAATGAGCGAGTGTAAATTACAACTACTTTCGACCGATGTCCTTCCGGTCTGCGCTTAATAAAAACTACCGCAATTGAGC
V N P P F V P T E N P T G C Y S L T F N V D E S W L Q E G Q T R I I F D G V N S

610 620 630 640 650 660 670 680 690 700 710 720

GCGTTTCATCTGTGGTGCAACGGGCGCTGGGTCGGTTACGGCCAGGACAGTCGTTTGCCGTCTGAATTTGACCTGAGCGCATTTTTACGCGCCGGAGAAAACCGCCTCGCGGTGATGGTG
CGCAAAGTAGACACCACGTTGCCCGCGACCCAGCCAATGCCGGTCCTGTCAGCAAACGGCAGACTTAAACTGGACTCGCGTAAAAATGCGCGGCCTCTTTTGGCGGAGCGCCACTACCAC
A F H L W C N G R W V G Y G Q D S R L P S E F D L S A F L R A G E N R L A V M V

730 740 750 760 770 780 790 800 810 820 830 840

CTGCGCTGGAGTGACGGCAGTTATCTGGAAGATCAGGATATGTGGCGGATGAGCGGCATTTTCCGTGACGTCTCGTTGCTGCATAAACCGACTACACAAATCAGCGATTTCCATGTTGCC
GACGCGACCTCACTGCCGTCAATAGACCTTCTAGTCCTATACACCGCCTACTCGCCGTAAAAGGCACTGCAGAGCAACGACGTATTTGGCTGATGTGTTTAGTCGCTAAAGGTACAACGG
L R W S D G S Y L E D Q D M W R M S G I F R D V S L L H K P T T Q I S D F H V A

850 860 870 880 890 900 910 920 930 940 950 960

ACTCGCTTTAATGATGATTTCAGCCGCGCTGTACTGGAGGCTGAAGTTCAGATGTGCGGCGAGTTGCGTGACTACCTACGGGTAACAGTTTCTTTATGGCAGGGTGAAACGCAGGTCGCC
TGAGCGAAATTACTACTAAAGTCGGCGCGACATGACCTCCGACTTCAAGTCTACACGCCGCTCAACGCACTGATGGATGCCCATTGTCAAAGAAATACCGTCCCACTTTGCGTCCAGCGG
T R F N D D F S R A V L E A E V Q M C G E L R D Y L R V T V S L W Q G E T Q V A
10 20 30 40 50 60 70 80 90 100 110 120

AGCGGCACCGCGCCTTTCGGCGGTGAAATTATCGATGAGCGTGGTGGTTATGCCGATCGCGTCACACTACGTCTGAACGTCGAAAACCCGAAACTGTGGAGCGCCGAAATCCCGAATCTC
TCGCCGTGGCGCGGAAAGCCGCCACTTTAATAGCTACTCGCACCACCAATACGGCTAGCGCAGTGTGATGCAGACTTGCAGCTTTTGGGCTTTGACACCTCGCGGCTTTAGGGCTTAGAG
S G T A P F G G E I I D E R G G Y A D R V T L R L N V E N P K L W S A E I P N L
130 140 150 160 170 180 190 200 210 220 230 240

TATCGTGCGGTGGTTGAACTGCACACCGCCGACGGCACGCTGATTGAAGCAGAAGCCTGCGATGTCGGTTTCCGCGAGGTGCGGATTGAAAATGGTCTGCTGCTGCTGAACGGCAAGCCG
ATAGCACGCCACCAACTTGACGTGTGGCGGCTGCCGTGCGACTAACTTCGTCTTCGGACGCTACAGCCAAAGGCGCTCCACGCCTAACTTTTACCAGACGACGACGACTTGCCGTTCGGC
Y R A V V E L H T A D G T L I E A E A C D V G F R E V R I E N G L L L L N G K P
250 260 270 280 290 300 310 320 330 340 350 360

TTGCTGATTCGAGGCGTTAACCGTCACGAGCATCATCCTCTGCATGGTCAGGTCATGGATGAGCAGACGATGGTGCAGGATATCCTGCTGATGAAGCAGAACAACTTTAACGCCGTGCGC
AACGACTAAGCTCCGCAATTGGCAGTGCTCGTAGTAGGAGACGTACCAGTCCAGTACCTACTCGTCTGCTACCACGTCCTATAGGACGACTACTTCGTCTTGTTGAAATTGCGGCACGCG
L L I R G V N R H E H H P L H G Q V M D E Q T M V Q D I L L M K Q N N F N A V R
370 380 390 400 410 420 430 440 450 460 470 480

TGTTCGCATTATCCGAACCATCCGCTGTGGTACACGCTGTGCGACCGCTACGGCCTGTATGTGGTGGATGAAGCCAATATTGAAACCCACGGCATGGTGCCAATGAATCGTCTGACCGAT
ACAAGCGTAATAGGCTTGGTAGGCGACACCATGTGCGACACGCTGGCGATGCCGGACATACACCACCTACTTCGGTTATAACTTTGGGTGCCGTACCACGGTTACTTAGCAGACTGGCTA
C S H Y P N H P L W Y T L C D R Y G L Y V V D E A N I E T H G M V P M N R L T D
490 500 510 520 530 540 550 560 570 580 590 600

GATCCGCGCTGGCTACCGGCGATGAGCGAACGCGTAACGCGAATGGTGCAGCGCGATCGTAATCACCCGAGTGTGATCATCTGGTCGCTGGGGAATGAATCAGGCCACGGCGCTAATCAC
CTAGGCGCGACCGATGGCCGCTACTCGCTTGCGCATTGCGCTTACCACGTCGCGCTAGCATTAGTGGGCTCACACTAGTAGACCAGCGACCCCTTACTTAGTCCGGTGCCGCGATTAGTG
D P R W L P A M S E R V T R M V Q R D R N H P S V I I W S L G N E S G H G A N H
610 620 630 640 650 660 670 680 690 700 710 720

GACGCGCTGTATCGCTGGATCAAATCTGTCGATCCTTCCCGCCCGGTGCAGTATGAAGGCGGCGGAGCCGACACCACGGCCACCGATATTATTTGCCCGATGTACGCGCGCGTGGATGAA
CTGCGCGACATAGCGACCTAGTTTAGACAGCTAGGAAGGGCGGGCCACGTCATACTTCCGCCGCCTCGGCTGTGGTGCCGGTGGCTATAATAAACGGGCTACATGCGCGCGCACCTACTT
D A L Y R W I K S V D P S R P V Q Y E G G G A D T T A T D I I C P M Y A R V D E
730 740 750 760 770 780 790 800 810 820 830 840

GACCAGCCCTTCCCGGCTGTGCCGAAATGGTCCATCAAAAAATGGCTTTCGCTACCTGGAGAGACGCGCCCGCTGATCCTTTGCGAATACGCCCACGCGATGGGTAACAGTCTTGGCGGT
CTGGTCGGGAAGGGCCGACACGGCTTTACCAGGTAGTTTTTTACCGAAAGCGATGGACCTCTCTGCGCGGGCGACTAGGAAACGCTTATGCGGGTGCGCTACCCATTGTCAGAACCGCCA
D Q P F P A V P K W S I K K W L S L P G E T R P L I L C E Y A H A M G N S L G G
850 860 870 880 890 900 910 920 930 940 950 960

TTCGCTAAATACTGGCAGGCGTTTCGTCAGTATCCCCGTTTACAGGGCGGCTTCGTCTGGGACTGGGTGGATCAGTCGCTGATTAAATATGATGAAAACGGCAACCCGTGGTCGGCTTAC
AAGCGATTTATGACCGTCCGCAAAGCAGTCATAGGGGCAAATGTCCCGCCGAAGCAGACCCTGACCCACCTAGTCAGCGACTAATTTATACTACTTTTGCCGTTGGGCACCAGCCGAATG
F A K Y W Q A F R Q Y P R L Q G G F V W D W V D Q S L I K Y D E N G N P W S A Y
970 980 990 1000 1010 1020 1030 1040 1050 1060 1070 1080

GGCGGTGATTTTGGCGATACGCCGAACGATCGCCAGTTCTGTATGAACGGTCTGGTCTTTGCCGACCGCACGCCGCATCCAGCGCTGACGGAAGCAAAACACCAGCAGCAGTTTTTCCAG
CCGCCACTAAAACCGCTATGCGGCTTGCTAGCGGTCAAGACATACTTGCCAGACCAGAAACGGCTGGCGTGCGGCGTAGGTCGCGACTGCCTTCGTTTTGTGGTCGTCGTCAAAAAGGTC
G G D F G D T P N D R Q F C M N G L V F A D R T P H P A L T E A K H Q Q Q F F Q
1090 1100 1110 1120 1130 1140 1150 1160 1170 1180 1190 1200

GenScript USA, Inc.
860 Centennial Ave. Piscataway, NJ 08854
Tel: 1-732-885-9188

TTCCGTTTATCCGGGCAAACCATCGAAGTGACCAGCGAATACCTGTTCCGTCATAGCGATAACGAGCTCCTGCACTGGATGGTGGCGCTGGATGGTAAGCCGCTGGCAAGCGGTGAAGTG
AAGGCAAATAGGCCCGTTTGGTAGCTTCACTGGTCGCTTATGGACAAGGCAGTATCGCTATTGCTCGAGGACGTGACCTACCACCGCGACCTACCATTCGGCGACCGTTCGCCACTTCAC
F R L S G Q T I E V T S E Y L F R H S D N E L L H W M V A L D G K P L A S G E V

10 20 30 40 50 60 70 80 90 100 110 120

CCTCTGGATGTCGCTCCACAAGGTAAACAGTTGATTGAACTGCCTGAACTACCGCAGCCGGAGAGCGCCGGGCAACTCTGGCTCACAGTACGCGTAGTGCAACCGAACGCGACCGCATGG
GGAGACCTACAGCGAGGTGTTCCATTTGTCAACTAACTTGACGGACTTGATGGCGTCGGCCTCTCGCGGCCCGTTGAGACCGAGTGTCATGCGCATCACGTTGGCTTGCGCTGGCGTACC
P L D V A P Q G K Q L I E L P E L P Q P E S A G Q L W L T V R V V Q P N A T A W

130 140 150 160 170 180 190 200 210 220 230 240

TCAGAAGCCGGGCACATCAGCGCCTGGCAGCAGTGGCGTCTGGCGGAAAACCTCAGTGTGACGCTCCCCGCCGCGTCCCACGCCATCCCGCATCTGACCACCAGCGAAATGGATTTTTGC
AGTCTTCGGCCCGTGTAGTCGCGGACCGTCGTCACCGCAGACCGCCTTTTGGAGTCACACTGCGAGGGGCGGCGCAGGGTGCGGTAGGGCGTAGACTGGTGGTCGCTTTACCTAAAAACG
S E A G H I S A W Q Q W R L A E N L S V T L P A A S H A I P H L T T S E M D F C

250 260 270 280 290 300 310 320 330 340 350 360

ATCGAGCTGGGTAATAAGCGTTGGCAATTTAACCGCCAGTCAGGCTTTCTTTCACAGATGTGGATTGGCGATAAAAAACAACTGCTGACGCCGCTGCGCGATCAGTTCACCCGTGCACCG
TAGCTCGACCCATTATTCGCAACCGTTAAATTGGCGGTCAGTCCGAAAGAAAGTGTCTACACCTAACCGCTATTTTTTGTTGACGACTGCGGCGACGCGCTAGTCAAGTGGGCACGTGGC
I E L G N K R W Q F N R Q S G F L S Q M W I G D K K Q L L T P L R D Q F T R A P

370 380 390 400 410 420 430 440 450 460 470 480

CTGGATAACGACATTGGCGTAAGTGAAGCGACCCGCATTGACCCTAACGCCTGGGTCGAACGCTGGAAGGCGGCGGGCCATTACCAGGCCGAAGCAGCGTTGTTGCAGTGCACGGCAGAT
GACCTATTGCTGTAACCGCATTCACTTCGCTGGGCGTAACTGGGATTGCGGACCCAGCTTGCGACCTTCCGCCGCCCGGTAATGGTCCGGCTTCGTCGCAACAACGTCACGTGCCGTCTA
L D N D I G V S E A T R I D P N A W V E R W K A A G H Y Q A E A A L L Q C T A D

490 500 510 520 530 540 550 560 570 580 590 600

ACACTTGCTGATGCGGTGCTGATTACGACCGCTCACGCGTGGCAGCATCAGGGGAAAACCTTATTTATCAGCCGGAAAACCTACCGGATTGATGGTAGTGGTCAAATGGCGATTACCGTT
TGTGAACGACTACGCCACGACTAATGCTGGCGAGTGCGCACCGTCGTAGTCCCCTTTTGGAATAAATAGTCGGCCTTTTGGATGGCCTAACTACCATCACCAGTTTACCGCTAATGGCAA
T L A D A V L I T T A H A W Q H Q G K T L F I S R K T Y R I D G S G Q M A I T V

610 620 630 640 650 660 670 680 690 700 710 720

GATGTTGAAGTGGCGAGCGATACACCGCATCCGGCGCGGATTGGCCTGAACTGCCAGCTGGCGCAGGTAGCAGAGCGGGTAAACTGGCTCGGATTAGGGCCGCAAGAAAACTATCCCGAC
CTACAACTTCACCGCTCGCTATGTGGCGTAGGCCGCGCCTAACCGGACTTGACGGTCGACCGCGTCCATCGTCTCGCCCATTTGACCGAGCCTAATCCCGGCGTTCTTTTGATAGGGCTG
D V E V A S D T P H P A R I G L N C Q L A Q V A E R V N W L G L G P Q E N Y P D

730 740 750 760 770 780 790 800 810 820 830 840

CGCCTTACTGCCGCCTGTTTTGACCGCTGGGATCTGCCATTGTCAGACATGTATACCCCGTACGTCTTCCCGAGCGAAAACGGTCTGCGCTGCGGGACGCGCGAATTGAATTATGGCCCA
GCGGAATGACGGCGGACAAAACTGGCGACCCTAGACGGTAACAGTCTGTACATATGGGGCATGCAGAAGGGCTCGCTTTTGCCAGACGCGACGCCCTGCGCGCTTAACTTAATACCGGGT
R L T A A C F D R W D L P L S D M Y T P Y V F P S E N G L R C G T R E L N Y G P

850 860 870 880 890 900 910 920 930 940 950 960

CACCAGTGGCGCGGCGACTTCCAGTTCAACATCAGCCGCTACAGTCAACAGCAACTGATGGAAACCAGCCATCGCCATCTGCTGCACGCGGAAGAAGGCACATGGCTGAATATCGACGGT
GTGGTCACCGCGCCGCTGAAGGTCAAGTTGTAGTCGGCGATGTCAGTTGTCGTTGACTACCTTTGGTCGGTAGCGGTAGACGACGTGCGCCTTCTTCCGTGTACCGACTTATAGCTGCCA
H Q W R G D F Q F N I S R Y S Q Q Q L M E T S H R H L L H A E E G T W L N I D G

970 980 990 1000 1010 1020 1030 1040 1050 1060 1070 1080

TTCCATATGGGGATTGGTGGCGACGACTCCTGGAGCCCGTCAGTATCGGCGGAATTCCAGCTGAGCGCCGGTCGCTACCATTACCAGTTGGTCTGGTGTCAAAAATAAGGTCGACAAGCT
AAGGTATACCCCTAACCACCGCTGCTGAGGACCTCGGGCAGTCATAGCCGCCTTAAGGTCGACTCGCGGCCAGCGATGGTAATGGTCAACCAGACCACAGTTTTTATTCCAGCTGTTCGA
F H M G I G G D D S W S P S V S A E F Q L S A G R Y H Y Q L V W C Q K *

1090 1100 1110 1120 1130 1140 1150 1160 1170 1180 1190 1200

TGCGGCCGCATAATGCTTAAGTCGAACAGAAAGTAATCGTATTGTACACGGCCGCATAATCGAAATTAATACGACTCACTATAGGGGAATTGTGAGCGGATAACAATTCCCCATCTTAGT
ACGCCGGCGTATTACGAATTCAGCTTGTCTTTCATTAGCATAACATGTGCCGGCGTATTAGCTTTAATTATGCTGAGTGATATCCCCTTAACACTCGCCTATTGTTAAGGGGTAGAATCA

DuetUP2 Primer
DuetDOWN1 Primer T7 promoter lac operator

10 20 30 40 50 60 70 80 90 100 110 120

ATATTAGTTAAGTATAAGAAGGAGATATACATATGGCAGATCTCAATTGGATATCGGCCGGCCACGCGATCGCTGACGTCGGTACCCTCGAGTCTGGTAAAGAAACCGCTGCTGCGAAAT
TATAATCAATTCATATTCTTCCTCTATATGTATACCGTCTAGAGTTAACCTATAGCCGGCCGGTGCGCTAGCGACTGCAGCCATGGGAGCTCAGACCATTTCTTTGGCGACGACGCTTTA

RBS MCS-2 M

K E T A A A K

S-Tag

130 140 150 160 170 180 190 200 210 220 230 240

TTGAACGCCAGCACATGGACTCGTCTACTAGCGCAGCTTAATTAACCTAGGCTGCTGCCACCGCTGAGCAATAACTAGCATAACCCCTTGGGGCCTCTAAACGGGTCTTGAGGGGTTTTT
AACTTGCGGTCGTGTACCTGAGCAGATGATCGCGTCGAATTAATTGGATCCGACGACGGTGGCGACTCGTTATTGATCGTATTGGGGAACCCCGGAGATTTGCCCAGAACTCCCCAAAAA

F E R Q H M D S

T7 Terminator Primer
T7 terminator

250 260 270 280 290 300 310 320 330 340 350 360

TGCTGAAAGGAGGAACTATATCCGGATTGGCGAATGGGACGCGCCCTGTAGCGGCGCATTAAGCGCGGCGGGTGTGGTGGTTACGCGCAGCGTGACCGCTACACTTGCCAGCGCCCTAGC
ACGACTTTCCTCCTTGATATAGGCCTAACCGCTTACCCTGCGCGGGACATCGCCGCGTAATTCGCGCCGCCCACACCACCAATGCGCGTCGCACTGGCGATGTGAACGGTCGCGGGATCG

f1 ori

370 380 390 400 410 420 430 440 450 460 470 480

GCCCGCTCCTTTCGCTTTCTTCCCTTCCTTTCTCGCCACGTTCGCCGGCTTTCCCCGTCAAGCTCTAAATCGGGGGCTCCCTTTAGGGTTCCGATTTAGTGCTTTACGGCACCTCGACCC
CGGGCGAGGAAAGCGAAAGAAGGGAAGGAAAGAGCGGTGCAAGCGGCCGAAAGGGGCAGTTCGAGATTTAGCCCCCGAGGGAAATCCCAAGGCTAAATCACGAAATGCCGTGGAGCTGGG

490 500 510 520 530 540 550 560 570 580 590 600

CAAAAAACTTGATTAGGGTGATGGTTCACGTAGTGGGCCATCGCCCTGATAGACGGTTTTTCGCCCTTTGACGTTGGAGTCCACGTTCTTTAATAGTGGACTCTTGTTCCAAACTGGAAC
GTTTTTTGAACTAATCCCACTACCAAGTGCATCACCCGGTAGCGGGACTATCTGCCAAAAAGCGGGAAACTGCAACCTCAGGTGCAAGAAATTATCACCTGAGAACAAGGTTTGACCTTG

610 620 630 640 650 660 670 680 690 700 710 720

AACACTCAACCCTATCTCGGTCTATTCTTTTGATTTATAAGGGATTTTGCCGATTTCGGCCTATTGGTTAAAAAATGAGCTGATTTAACAAAAATTTAACGCGAATTTTAACAAAATATT
TTGTGAGTTGGGATAGAGCCAGATAAGAAAACTAAATATTCCCTAAAACGGCTAAAGCCGGATAACCAATTTTTTACTCGACTAAATTGTTTTTAAATTGCGCTTAAAATTGTTTTATAA

730 740 750 760 770 780 790 800 810 820 830 840

AACGTTTACAATTTCTGGCGGCACGATGGCATGAGATTATCAAAAAGGATCTTCACCTAGATCCTTTTAAATTAAAAATGAAGTTTTAAATCAATCTAAAGTATATATGAGTAAACTTGG
TTGCAAATGTTAAAGACCGCCGTGCTACCGTACTCTAATAGTTTTTCCTAGAAGTGGATCTAGGAAAATTTAATTTTTACTTCAAAATTTAGTTAGATTTCATATATACTCATTTGAACC

850 860 870 880 890 900 910 920 930 940 950 960

TCTGACAGTTACCAATGCTTAATCAGTGAGGCACCTATCTCAGCGATCTGTCTATTTCGTTCATCCATAGTTGCCTGACTCCCCGTCGTGTAGATAACTACGATACGGGAGGGCTTACCA
AGACTGTCAATGGTTACGAATTAGTCACTCCGTGGATAGAGTCGCTAGACAGATAAAGCAAGTAGGTATCAACGGACTGAGGGGCAGCACATCTATTGATGCTATGCCCTCCCGAATGGT

* W H K I L S A G I E A I Q R N R E D M T A Q S G T T Y I V V I R S P K G

AmpR

970 980 990 1000 1010 1020 1030 1040 1050 1060 1070 1080

TCTGGCCCCAGTGCTGCAATGATACCGCGAGACCCACGCTCACCGGCTCCAGATTTATCAGCAATAAACCAGCCAGCCGGAAGGGCCGAGCGCAGAAGTGGTCCTGCAACTTTATCCGCC
AGACCGGGGTCACGACGTTACTATGGCGCTCTGGGTGCGAGTGGCCGAGGTCTAAATAGTCGTTATTTGGTCGGTCGGCCTTCCCGGCTCGCGTCTTCACCAGGACGTTGAAATAGGCGG

D P G L A A I I G R S G R E G A G S K D A I F W G A P L A S R L L P G A V K D A

1090 1100 1110 1120 1130 1140 1150 1160 1170 1180 1190 1200

TCCATCCAGTCTATTAATTGTTGCCGGGAAGCTAGAGTAAGTAGTTCGCCAGTTAATAGTTTGCGCAACGTTGTTGCCATTGCTACAGGCATCGTGGTGTCACGCTCGTCGTTTGGTATG
AGGTAGGTCAGATAATTAACAACGGCCCTTCGATCTCATTCATCAAGCGGTCAATTATCAAACGCGTTGCAACAACGGTAACGATGTCCGTAGCACCACAGTGCGAGCAGCAAACCATAC
E M W D I L Q Q R S A L T L L E G T L L K R L T T A M A V P M T T D R E D N P I

10 20 30 40 50 60 70 80 90 100 110 120

GCTTCATTCAGCTCCGGTTCCCAACGATCAAGGCGAGTTACATGATCCCCCATGTTGTGCAAAAAAGCGGTTAGCTCCTTCGGTCCTCCGATCGTTGTCAGAAGTAAGTTGGCCGCAGTG
CGAAGTAAGTCGAGGCCAAGGGTTGCTAGTTCCGCTCAATGTACTAGGGGGTACAACACGTTTTTTCGCCAATCGAGGAAGCCAGGAGGCTAGCAACAGTCTTCATTCAACCGGCGTCAC
A E N L E P E W R D L R T V H D G M N H L F A T L E K P G G I T T L L L N A A T

130 140 150 160 170 180 190 200 210 220 230 240

TTATCACTCATGGTTATGGCAGCACTGCATAATTCTCTTACTGTCATGCCATCCGTAAGATGCTTTTCTGTGACTGGTGAGTACTCAACCAAGTCATTCTGAGAATAGTGTATGCGGCGA
AATAGTGAGTACCAATACCGTCGTGACGTATTAAGAGAATGACAGTACGGTAGGCATTCTACGAAAAGACACTGACCACTCATGAGTTGGTTCAGTAAGACTCTTATCACATACGCCGCT
N D S M T I A A S C L E R V T M G D T L H K E T V P S Y E V L D N Q S Y H I R R

250 260 270 280 290 300 310 320 330 340 350 360

CCGAGTTGCTCTTGCCCGGCGTCAATACGGGATAATACCGCGCCACATAGCAGAACTTTAAAAGTGCTCATCATTGGAAAACGTTCTTCGGGGCGAAAACTCTCAAGGATCTTACCGCTG
GGCTCAACGAGAACGGGCCGCAGTTATGCCCTATTATGGCGCGGTGTATCGTCTTGAAATTTTCACGAGTAGTAACCTTTTGCAAGAAGCCCCGCTTTTGAGAGTTCCTAGAATGGCGAC
G L Q E Q G A D I R S L V A G C L L V K F T S M M P F R E E P R F S E L I K G S

370 380 390 400 410 420 430 440 450 460 470 480

TTGAGATCCAGTTCGATGTAACCCACTCGTGCACCCAACTGATCTTCAGCATCTTTTACTTTCACCAGCGTTTCTGGGTGAGCAAAAACAGGAAGGCAAAATGCCGCAAAAAAGGGAATA
AACTCTAGGTCAAGCTACATTGGGTGAGCACGTGGGTTGACTAGAAGTCGTAGAAAATGAAAGTGGTCGCAAAGACCCACTCGTTTTTGTCCTTCCGTTTTACGGCGTTTTTTCCCTTAT
N L D L E I Y G V R A G L Q D E A D K V K V L T E P H A F V P L C F A A F F P I

490 500 510 520 530 540 550 560 570 580 590 600

AGGGCGACACGGAAATGTTGAATACTCATACTCTTCCTTTTTCAATCATGATTGAAGCATTTATCAGGGTTATTGTCTCATGAGCGGATACATATTTGAATGTATTTAGAAAAATAAACA
TCCCGCTGTGCCTTTACAACTTATGAGTATGAGAAGGAAAAAGTTAGTACTAACTTCGTAAATAGTCCCAATAACAGAGTACTCGCCTATGTATAAACTTACATAAATCTTTTTATTTGT
L A V R F H Q I S M

AmpR promoter

610 620 630 640 650 660 670 680 690 700 710 720

AATAGGTCATGACCAAAATCCCTTAACGTGAGTTTTCGTTCCACTGAGCGTCAGACCCCGTAGAAAAGATCAAAGGATCTTCTTGAGATCCTTTTTTTCTGCGCGTAATCTGCTGCTTGC
TTATCCAGTACTGGTTTTAGGGAATTGCACTCAAAAGCAAGGTGACTCGCAGTCTGGGGCATCTTTTCTAGTTTCCTAGAAGAACTCTAGGAAAAAAAGACGCGCATTAGACGACGAACG

ori

730 740 750 760 770 780 790 800 810 820 830 840

AAACAAAAAAACCACCGCTACCAGCGGTGGTTTGTTTGCCGGATCAAGAGCTACCAACTCTTTTTCCGAAGGTAACTGGCTTCAGCAGAGCGCAGATACCAAATACTGTCCTTCTAGTGT
TTTGTTTTTTTGGTGGCGATGGTCGCCACCAAACAAACGGCCTAGTTCTCGATGGTTGAGAAAAAGGCTTCCATTGACCGAAGTCGTCTCGCGTCTATGGTTTATGACAGGAAGATCACA

850 860 870 880 890 900 910 920 930 940 950 960

AGCCGTAGTTAGGCCACCACTTCAAGAACTCTGTAGCACCGCCTACATACCTCGCTCTGCTAATCCTGTTACCAGTGGCTGCTGCCAGTGGCGATAAGTCGTGTCTTACCGGGTTGGACT
TCGGCATCAATCCGGTGGTGAAGTTCTTGAGACATCGTGGCGGATGTATGGAGCGAGACGATTAGGACAATGGTCACCGACGACGGTCACCGCTATTCAGCACAGAATGGCCCAACCTGA

970 980 990 1000 1010 1020 1030 1040 1050 1060 1070 1080

CAAGACGATAGTTACCGGATAAGGCGCAGCGGTCGGGCTGAACGGGGGGTTCGTGCACACAGCCCAGCTTGGAGCGAACGACCTACACCGAACTGAGATACCTACAGCGTGAGCTATGAG
GTTCTGCTATCAATGGCCTATTCCGCGTCGCCAGCCCGACTTGCCCCCCAAGCACGTGTGTCGGGTCGAACCTCGCTTGCTGGATGTGGCTTGACTCTATGGATGTCGCACTCGATACTC

1090 1100 1110 1120 1130 1140 1150 1160 1170 1180 1190 1200

AAAGCGCCACGCTTCCCGAAGGGAGAAAGGCGGACAGGTATCCGGTAAGCGGCAGGGTCGGAACAGGAGAGCGCACGAGGGAGCTTCCAGGGGGAAACGCCTGGTATCTTTATAGTCCTG
TTTCGCGGTGCGAAGGGCTTCCCTCTTTCCGCCTGTCCATAGGCCATTCGCCGTCCCAGCCTTGTCCTCTCGCGTGCTCCCTCGAAGGTCCCCCTTTGCGGACCATAGAAATATCAGGAC

10 20 30 40 50 60 70 80 90 100 110 120

TCGGGTTTCGCCACCTCTGACTTGAGCGTCGATTTTTGTGATGCTCGTCAGGGGGGCGGAGCCTATGGAAAAACGCCAGCAACGCGGCCTTTTTACGGTTCCTGGCCTTTTGCTGGCCTT
AGCCCAAAGCGGTGGAGACTGAACTCGCAGCTAAAAACACTACGAGCAGTCCCCCCGCCTCGGATACCTTTTTGCGGTCGTTGCGCCGGAAAAATGCCAAGGACCGGAAAACGACCGGAA

130 140 150 160 170 180 190 200 210 220 230 240

TTGCTCACATGTTCTTTCCTGCGTTATCCCCTGATTCTGTGGATAACCGTATTACCGCCTTTGAGTGAGCTGATACCGCTCGCCGCAGCCGAACGACCGAGCGCAGCGAGTCAGTGAGCG
AACGAGTGTACAAGAAAGGACGCAATAGGGGACTAAGACACCTATTGGCATAATGGCGGAAACTCACTCGACTATGGCGAGCGGCGTCGGCTTGCTGGCTCGCGTCGCTCAGTCACTCGC

250 260 270 280 290 300 310 320 330 340 350 360

AGGAAGCGGAAGAGCGCCTGATGCGGTATTTTCTCCTTACGCATCTGTGCGGTATTTCACACCGCATATATGGTGCACTCTCAGTACAATCTGCTCTGATGCCGCATAGTTAAGCCAGTA
TCCTTCGCCTTCTCGCGGACTACGCCATAAAAGAGGAATGCGTAGACACGCCATAAAGTGTGGCGTATATACCACGTGAGAGTCATGTTAGACGAGACTACGGCGTATCAATTCGGTCAT

370 380 390 400 410 420 430 440 450 460 470 480

TACACTCCGCTATCGCTACGTGACTGGGTCATGGCTGCGCCCCGACACCCGCCAACACCCGCTGACGCGCCCTGACGGGCTTGTCTGCTCCCGGCATCCGCTTACAGACAAGCTGTGACC
ATGTGAGGCGATAGCGATGCACTGACCCAGTACCGACGCGGGGCTGTGGGCGGTTGTGGGCGACTGCGCGGGACTGCCCGAACAGACGAGGGCCGTAGGCGAATGTCTGTTCGACACTGG

490 500 510 520 530 540 550 560 570 580 590 600

GTCTCCGGGAGCTGCATGTGTCAGAGGTTTTCACCGTCATCACCGAAACGCGCGAGGCAGCTGCGGTAAAGCTCATCAGCGTGGTCGTGAAGCGATTCACAGATGTCTGCCTGTTCATCC
CAGAGGCCCTCGACGTACACAGTCTCCAAAAGTGGCAGTAGTGGCTTTGCGCGCTCCGTCGACGCCATTTCGAGTAGTCGCACCAGCACTTCGCTAAGTGTCTACAGACGGACAAGTAGG
* L N E G D D G F R A L C S R Y L E D A H D H L S E C I D A Q E D
rop

610 620 630 640 650 660 670 680 690 700 710 720

GCGTCCAGCTCGTTGAGTTTCTCCAGAAGCGTTAATGTCTGGCTTCTGATAAAGCGGGCCATGTTAAGGGCGGTTTTTTCCTGTTTGGTCACTGATGCCTCCGTGTAAGGGGGATTTCTG
CGCAGGTCGAGCAACTCAAAGAGGTCTTCGCAATTACAGACCGAAGACTATTTCGCCCGGTACAATTCCCGCCAAAAAAGGACAAACCAGTGACTACGGAGGCACATTCCCCCTAAAGAC
A D L E N L K E L L T L T Q S R I F R A M N L A T K E Q K T V

730 740 750 760 770 780 790 800 810 820 830 840

TTCATGGGGGTAATGATACCGATGAAACGAGAGAGGATGCTCACGATACGGGTTACTGATGATGAACATGCCCGGTTACTGGAACGTTGTGAGGGTAAACAACTGGCGGTATGGATGCGG
AAGTACCCCCATTACTATGGCTACTTTGCTCTCTCCTACGAGTGCTATGCCCAATGACTACTACTTGTACGGGCCAATGACCTTGCAACACTCCCATTTGTTGACCGCCATACCTACGCC

850 860 870 880 890 900 910 920 930 940 950 960

CGGGACCAGAGAAAAATCACTCAGGGTCAATGCCAGCGCTTCGTTAATACAGATGTAGGTGTTCCACAGGGTAGCCAGCAGCATCCTGCGATGCAGATCCGGAACATAATGGTGCAGGGC
GCCCTGGTCTCTTTTTAGTGAGTCCCAGTTACGGTCGCGAAGCAATTATGTCTACATCCACAAGGTGTCCCATCGGTCGTCGTAGGACGCTACGTCTAGGCCTTGTATTACCACGTCCCG

970 980 990 1000 1010 1020 1030 1040 1050 1060 1070 1080

GCTGACTTCCGCGTTTCCAGACTTTACGAAACACGGAAACCGAAGACCATTCATGTTGTTGCTCAGGTCGCAGACGTTTTGCAGCAGCAGTCGCTTCACGTTCGCTCGCGTATCGGTGAT
CGACTGAAGGCGCAAAGGTCTGAAATGCTTTGTGCCTTTGGCTTCTGGTAAGTACAACAACGAGTCCAGCGTCTGCAAAACGTCGTCGTCAGCGAAGTGCAAGCGAGCGCATAGCCACTA

1090 1100 1110 1120 1130 1140 1150 1160 1170 1180 1190 1200

TCATTCTGCTAACCAGTAAGGCAACCCCGCCAGCCTAGCCGGGTCCTCAACGACAGGAGCACGATCATGCTAGTCATGCCCCGCGCCCACCGGAAGGAGCTGACTGGGTTGAAGGCTCTC
AGTAAGACGATTGGTCATTCCGTTGGGGCGGTCGGATCGGCCCAGGAGTTGCTGTCCTCGTGCTAGTACGATCAGTACGGGGCGCGGGTGGCCTTCCTCGACTGACCCAACTTCCGAGAG

1210 1220 1230 1240 1250 1260 1270 1280 1290 1300 1310 1320

AAGGGCATCGGTCGAGATCCCGGTGCCTAATGAGTGAGCTAACTTACATTAATTGCGTTGCGCTCACTGCCCGCTTTCCAGTCGGGAAACCTGTCGTGCCAGCTGCATTAATGAATCGGC
TTCCCGTAGCCAGCTCTAGGGCCACGGATTACTCACTCGATTGAATGTAATTAACGCAACGCGAGTGACGGGCGAAAGGTCAGCCCTTTGGACAGCACGGTCGACGTAATTACTTAGCCG
* Q G S E L R S V Q R A L Q M L S D A
lacI

1330 1340 1350 1360 1370 1380 1390 1400 1410 1420 1430 1440

CAACGCGCGGGGAGAGGCGGTTTGCGTATTGGGCGCCAGGGTGGTTTTTCTTTTCACCAGTGAGACGGGCAACAGCTGATTGCCCTTCACCGCCTGGCCCTGAGAGAGTTGCAGCAAGCG
GTTGCGCGCCCCTCTCCGCCAAACGCATAACCCGCGGTCCCACCAAAAAGAAAAGTGGTCACTCTGCCCGTTGTCGACTAACGGGAAGTGGCGGACCGGGACTCTCTCAACGTCGTTCGC
L A R P S A T Q T N P A L T T K R K V L S V P L L Q N G K V A Q G G Q S L Q L L R
10 20 30 40 50 60 70 80 90 100 110 120

GTCCACGCTGGTTTGCCCCAGCAGGCGAAAATCCTGTTTGATGGTGGTTAACGGCGGGATATAACATGAGCTGTCTTCGGTATCGTCGTATCCCACTACCGAGATGTCCGCACCAACGCG
CAGGTGCGACCAAACGGGGTCGTCCGCTTTTAGGACAAACTACCACCAATTGCCGCCCTATATTGTACTCGACAGAAGCCATAGCAGCATAGGGTGATGGCTCTACAGGCGTGGTTGCGC
D V S T Q G L L R F D Q K I T T L P P I Y C S S D E T D D Y G V V S I D A G V R
130 140 150 160 170 180 190 200 210 220 230 240

CAGCCCGGACTCGGTAATGGCGCGCATTGCGCCCAGCGCCATCTGATCGTTGGCAACCAGCATCGCAGTGGGAACGATGCCCTCATTCAGCATTTGCATGGTTTGTTGAAAACCGGACAT
GTCGGGCCTGAGCCATTACCGCGCGTAACGCGGGTCGCGGTAGACTAGCAACCGTTGGTCGTAGCGTCACCCTTGCTACGGGAGTAAGTCGTAAACGTACCAAACAACTTTTGGCCTGTA
L G S E T I A R M A G L A M Q D N A V L M A T P V I G E N L M Q M T Q Q F G S M
250 260 270 280 290 300 310 320 330 340 350 360

GGCACTCCAGTCGCCTTCCCGTTCCGCTATCGGCTGAATTTGATTGCGAGTGAGATATTTATGCCAGCCAGCCAGACGCAGACGCGCCGAGACAGAACTTAATGGGCCCGCTAACAGCGC
CCGTGAGGTCAGCGGAAGGGCAAGGCGATAGCCGACTTAAACTAACGCTCACTCTATAAATACGGTCGGTCGGTCTGCGTCTGCGCGGCTCTGTCTTGAATTACCCGGGCGATTGTCGCG
A S W D G E R E A I P Q I Q N R T L Y K H W G A L R L R A S V S S L P G A L L A
370 380 390 400 410 420 430 440 450 460 470 480

GATTTGCTGGTGACCCAATGCGACCAGATGCTCCACGCCCAGTCGCGTACCGTCTTCATGGGAGAAAATAATACTGTTGATGGGTGTCTGGTCAGAGACATCAAGAAATAACGCCGGAAC
CTAAACGACCACTGGGTTACGCTGGTCTACGAGGTGCGGGTCAGCGCATGGCAGAAGTACCCTCTTTTATTATGACAACTACCCACAGACCAGTCTCTGTAGTTCTTTATTGCGGCCTTG
I Q Q H G L A V L H E V G L R T G D E H S F I I S N I P T Q D S V D L F L A P V
490 500 510 520 530 540 550 560 570 580 590 600

ATTAGTGCAGGCAGCTTCCACAGCAATGGCATCCTGGTCATCCAGCGGATAGTTAATGATCAGCCCACTGACGCGTTGCGCGAGAAGATTGTGCACCGCCGCTTTACAGGCTTCGACGCC
TAATCACGTCCGTCGAAGGTGTCGTTACCGTAGGACCAGTAGGTCGCCTATCAATTACTAGTCGGGTGACTGCGCAACGCGCTCTTCTAACACGTGGCGGCGAAATGTCCGAAGCTGCGG
N T C A A E V A I A D Q D D L P Y N I I L G S V R Q A L L N H V A A K C A E V G
610 620 630 640 650 660 670 680 690 700 710 720

GCTTCGTTCTACCATCGACACCACCACGCTGGCACCCAGTTGATCGGCGCGAGATTTAATCGCCGCGACAATTTGCGACGGCGCGTGCAGGGCCAGACTGGAGGTGGCAACGCCAATCAG
CGAAGCAAGATGGTAGCTGTGGTGGTGCGACCGTGGGTCAACTAGCCGCGCTCTAAATTAGCGGCGCTGTTAAACGCTGCCGCGCACGTCCCGGTCTGACCTCCACCGTTGCGGTTAGTC
S R E V M S V V V S A G L Q D A R S K I A A V I Q S P A H L A L S S T A V G I L
730 740 750 760 770 780 790 800 810 820 830 840

CAACGACTGTTTGCCCGCCAGTTGTTGTGCCACGCGGTTGGGAATGTAATTCAGCTCCGCCATCGCCGCTTCCACTTTTTCCCGCGTTTTCGCAGAAACGTGGCTGGCCTGGTTCACCAC
GTTGCTGACAAACGGGCGGTCAACAACACGGTGCGCCAACCCTTACATTAAGTCGAGGCGGTAGCGGCGAAGGTGAAAAAGGGCGCAAAAGCGTCTTTGCACCGACCGGACCAAGTGGTG
L S Q K G A L Q Q A V R N P I Y N L E A M A A E V K E R T K A S V H S A Q N V V
850 860 870 880 890 900 910 920 930 940 950 960

GCGGGAAACGGTCTGATAAGAGACACCGGCATACTCTGCGACATCGTATAACGTTACTGGTTTCACATTCACCACCCTGAATTGACTCTCTTCCGGGCGCTATCATGCCATACCGCGAAA
CGCCCTTTGCCAGACTATTCTCTGTGGCCGTATGAGACGCTGTAGCATATTGCAATGACCAAAGTGTAAGTGGTGGGACTTAACTGAGAGAAGGCCCGCGATAGTACGGTATGGCGCTTT
R S V T Q Y S V G A Y E A V D Y L T V P K V
lacI promoter
970 980 990 1000 1010 1020 1030 1040 1050 1060 1070 1080

GGTTTTGCGCCATTCGATGGTGTCCGGGATCTCGACGCTCTCCCTTATGCGACTCCTGCATTAGGAAGCAGCCCAGTAGTAGGTTGAGGCCGTTGAGCACCGCCGCCGCAAGGAATGGTG
CCAAAACGCGGTAAGCTACCACAGGCCCTAGAGCTGCGAGAGGGAATACGCTGAGGACGTAATCCTTCGTCGGGTCATCATCCAACTCCGGCAACTCGTGGCGGCGGCGTTCCTTACCAC
1090 1100 1110 1120 1130 1140 1150 1160 1170 1180 1190 1200

CATGCAAGGAGATGGCGCCCAACAGTCCCCCGGCCACGGGGCCTGCCACCATACCCACGCCGAAACAAGCGCTCATGAGCCCGAAGTGGCGAGCCCGATCTTCCCCATCGGTGATGTCGG
GTACGTTCCTCTACCGCGGGTTGTCAGGGGGCCGGTGCCCCGGACGGTGGTATGGGTGCGGCTTTGTTCGCGAGTACTCGGGCTTCACCGCTCGGGCTAGAAGGGGTAGCCACTACAGCC

10 20 30 40 50 60 70 80 90 100 110 120

CGATATAGGCGCCAGCAACCGCACCTGTGGCGCCGGTGATGCCGGCCACGATGCGTCCGGCGTAGAGGATCGAGATCGATCTCGATCCCGCGAAATTAATACGACTCACTATAGG
GCTATATCCGCGGTCGTTGGCGTGGACACCGCGGCCACTACGGCCGGTGCTACGCAGGCCGCATCTCCTAGCTCTAGCTAGAGCTAGGGCGCTTTAATTATGCTGAGTGATATCC

pET Upstream Primer
T7 promoter

130 140 150 160 170 180 190 200 210 220 230 240
