# Supplementary material for: An engineered genetic circuit for lactose intolerance alleviation
Source: BMC Biol. 2021 Jul 5;19:137. doi: 10.1186/s12915-021-01070-9 (PMC8259030; doi:10.1186/s12915-021-01070-9)
Supplement: Supplementary file 4 — Additional file 4 The plasmid profile of pETDuet1-0. [file 12915_2021_1070_MOESM4_ESM.pdf]

Created by GenSmart Design, GenScript

Created time: 08:51:32, 03/26/2020

1. Map

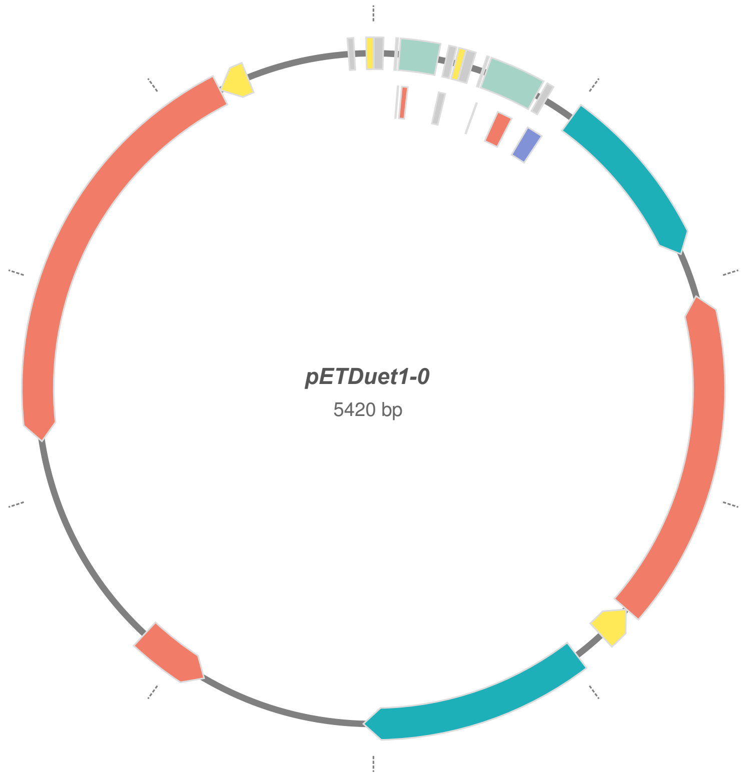

2. Construct Information

Construct name	*pETDuet1-0*
Construct size (bp)	5420 bp

3. Sequence

GGAATTGTGAGCGGATAACAATTCCCCTCTAGAAATAATTTTGTTTAACTTTAAGAAGGAGATATACCATGGGCAGCAGCCATCACCATCATCACCACAGCCAGGATCCGAATTCGAGCT
CCTTAACACTCGCCTATTGTTAAGGGGAGATCTTTATTAAAACAAATTGAAATTCTTCCTCTATATGGTACCCGTCGTCGGTAGTGGTAGTAGTGGTGTCGGTCCTAGGCTTAAGCTCGA

lac operator · RBS · MCS-1 · M

H H H H H H

6xHis

10 20 30 40 50 60 70 80 90 100 110 120

CGGCGCGCCTGCAGGTCGACAAGCTTGCGGCCGCATAATGCTTAAGTCGAACAGAAAGTAATCGTATTGTACACGGCCGCATAATCGAAATTAATACGACTCACTATAGGGGAATTGTGA
GCCGCGCGGACGTCCAGCTGTTCGAACGCCGGCGTATTACGAATTCAGCTTGTCTTTCATTAGCATAACATGTGCCGGCGTATTAGCTTTAATTATGCTGAGTGATATCCCCTTAACACT

DuetUP2 Primer

DuetDOWN1 Primer · T7 promoter · lac operator

130 140 150 160 170 180 190 200 210 220 230 240

GCGGATAACAATTCCCCATCTTAGTATATTAGTTAAGTATAAGAAGGAGATATACATATGGCAGATCTCAATTGGATATCGGCCGGCCACGCGATCGCTGACGTCGGTACCCTCGAGTCT
CGCCTATTGTTAAGGGGTAGAATCATATAATCAATTCATATTCTTCCTCTATATGTATACCGTCTAGAGTTAACCTATAGCCGGCCGGTGCGCTAGCGACTGCAGCCATGGGAGCTCAGA

RBS · MCS-2 · M

250 260 270 280 290 300 310 320 330 340 350 360

GGTAAAGAAACCGCTGCTGCGAAATTTGAACGCCAGCACATGGACTCGTCTACTAGCGCAGCTTAATTAACCTAGGCTGCTGCCACCGCTGAGCAATAACTAGCATAACCCCTTGGGGCC
CCATTTCTTTGGCGACGACGCTTTAAACTTGCGGTCGTGTACCTGAGCAGATGATCGCGTCGAATTAATTGGATCCGACGACGGTGGCGACTCGTTATTGATCGTATTGGGGAACCCCGG

K E T A A A K F E R Q H M D S

S-Tag · T7 Terminator Primer

T7 terminator

370 380 390 400 410 420 430 440 450 460 470 480

TCTAAACGGGTCTTGAGGGGTTTTTTGCTGAAAGGAGGAACTATATCCGGATTGGCGAATGGGACGCGCCCTGTAGCGGCGCATTAAGCGCGGCGGGTGTGGTGGTTACGCGCAGCGTGA
AGATTTGCCCAGAACTCCCCAAAAAACGACTTTCCTCCTTGATATAGGCCTAACCGCTTACCCTGCGCGGGACATCGCCGCGTAATTCGCGCCGCCCACACCACCAATGCGCGTCGCACT

f1 ori

490 500 510 520 530 540 550 560 570 580 590 600

CCGCTACACTTGCCAGCGCCCTAGCGCCCGCTCCTTTCGCTTTCTTCCCTTCCTTTCTCGCCACGTTCGCCGGCTTTCCCCGTCAAGCTCTAAATCGGGGGCTCCCTTTAGGGTTCCGAT
GGCGATGTGAACGGTCGCGGGATCGCGGGCGAGGAAAGCGAAAGAAGGGAAGGAAAGAGCGGTGCAAGCGGCCGAAAGGGGCAGTTCGAGATTTAGCCCCCGAGGGAAATCCCAAGGCTA

610 620 630 640 650 660 670 680 690 700 710 720

TTAGTGCTTTACGGCACCTCGACCCCAAAAAACTTGATTAGGGTGATGGTTCACGTAGTGGGCCATCGCCCTGATAGACGGTTTTTCGCCCTTTGACGTTGGAGTCCACGTTCTTTAATA
AATCACGAAATGCCGTGGAGCTGGGGTTTTTTGAACTAATCCCACTACCAAGTGCATCACCCGGTAGCGGGACTATCTGCCAAAAAGCGGGAAACTGCAACCTCAGGTGCAAGAAATTAT

730 740 750 760 770 780 790 800 810 820 830 840

GTGGACTCTTGTTCCAAACTGGAACAACACTCAACCCTATCTCGGTCTATTCTTTTGATTTATAAGGGATTTTGCCGATTTCGGCCTATTGGTTAAAAAATGAGCTGATTTAACAAAAAT
CACCTGAGAACAAGGTTTGACCTTGTTGTGAGTTGGGATAGAGCCAGATAAGAAAACTAAATATTCCCTAAAACGGCTAAAGCCGGATAACCAATTTTTTACTCGACTAAATTGTTTTTA

850 860 870 880 890 900 910 920 930 940 950 960

TTAACGCGAATTTTAACAAAATATTAACGTTTACAATTTCTGGCGGCACGATGGCATGAGATTATCAAAAAGGATCTTCACCTAGATCCTTTTAAATTAAAAATGAAGTTTTAAATCAAT
AATTGCGCTTAAAATTGTTTTATAATTGCAAATGTTAAAGACCGCCGTGCTACCGTACTCTAATAGTTTTTCCTAGAAGTGGATCTAGGAAAATTTAATTTTTACTTCAAAATTTAGTTA

970 980 990 1000 1010 1020 1030 1040 1050 1060 1070 1080

CTAAAGTATATATGAGTAAACTTGGTCTGACAGTTACCAATGCTTAATCAGTGAGGCACCTATCTCAGCGATCTGTCTATTTCGTTCATCCATAGTTGCCTGACTCCCCGTCGTGTAGAT
GATTTCATATATACTCATTTGAACCAGACTGTCAATGGTTACGAATTAGTCACTCCGTGGATAGAGTCGCTAGACAGATAAAGCAAGTAGGTATCAACGGACTGAGGGGCAGCACATCTA

* W H K I L S A G I E A I Q R N R E D M T A Q S G T T Y I

AmpR

1090 1100 1110 1120 1130 1140 1150 1160 1170 1180 1190 1200

AACTACGATACGGGAGGGCTTACCATCTGGCCCCAGTGCTGCAATGATACCGCGAGACCCACGCTCACCGGCTCCAGATTTATCAGCAATAAACCAGCCAGCCGGAAGGGCCGAGCGCAG
TTGATGCTATGCCCTCCCGAATGGTAGACCGGGGTCACGACGTTACTATGGCGCTCTGGGTGCGAGTGGCCGAGGTCTAAATAGTCGTTATTTGGTCGGTCGGCCTTCCCGGCTCGCGTC
V V I R S P K G D P G L A A I I G R S G R E G A G S K D A I F W G A P L A S R L
10 20 30 40 50 60 70 80 90 100 110 120

AAGTGGTCCTGCAACTTTATCCGCCTCCATCCAGTCTATTAATTGTTGCCGGGAAGCTAGAGTAAGTAGTTCGCCAGTTAATAGTTTGCGCAACGTTGTTGCCATTGCTACAGGCATCGT
TTCACCAGGACGTTGAAATAGGCGGAGGTAGGTCAGATAATTAACAACGGCCCTTCGATCTCATTCATCAAGCGGTCAATTATCAAACGCGTTGCAACAACGGTAACGATGTCCGTAGCA
L P G A V K D A E M W D I L Q Q R S A L T L L E G T L L K R L T T A M A V P M T
130 140 150 160 170 180 190 200 210 220 230 240

GGTGTCACGCTCGTCGTTTGGTATGGCTTCATTCAGCTCCGGTTCCCAACGATCAAGGCGAGTTACATGATCCCCCATGTTGTGCAAAAAAGCGGTTAGCTCCTTCGGTCCTCCGATCGT
CCACAGTGCGAGCAGCAAACCATACCGAAGTAAGTCGAGGCCAAGGGTTGCTAGTTCCGCTCAATGTACTAGGGGGTACAACACGTTTTTTCGCCAATCGAGGAAGCCAGGAGGCTAGCA
T D R E D N P I A E N L E P E W R D L R T V H D G M N H L F A T L E K P G G I T
250 260 270 280 290 300 310 320 330 340 350 360

TGTCAGAAGTAAGTTGGCCGCAGTGTTATCACTCATGGTTATGGCAGCACTGCATAATTCTCTTACTGTCATGCCATCCGTAAGATGCTTTTCTGTGACTGGTGAGTACTCAACCAAGTC
ACAGTCTTCATTCAACCGGCGTCACAATAGTGAGTACCAATACCGTCGTGACGTATTAAGAGAATGACAGTACGGTAGGCATTCTACGAAAAGACACTGACCACTCATGAGTTGGTTCAG
T L L L N A A T N D S M T I A A S C L E R V T M G D T L H K E T V P S Y E V L D
370 380 390 400 410 420 430 440 450 460 470 480

ATTCTGAGAATAGTGTATGCGGCGACCGAGTTGCTCTTGCCCGGCGTCAATACGGGATAATACCGCGCCACATAGCAGAACTTTAAAAGTGCTCATCATTGGAAAACGTTCTTCGGGGCG
TAAGACTCTTATCACATACGCCGCTGGCTCAACGAGAACGGGCCGCAGTTATGCCCTATTATGGCGCGGTGTATCGTCTTGAAATTTTCACGAGTAGTAACCTTTTGCAAGAAGCCCCGC
N Q S Y H I R R G L Q E Q G A D I R S L V A G C L L V K F T S M M P F R E E P R
490 500 510 520 530 540 550 560 570 580 590 600

AAAACTCTCAAGGATCTTACCGCTGTTGAGATCCAGTTCGATGTAACCCACTCGTGCACCCAACTGATCTTCAGCATCTTTTACTTTCACCAGCGTTTCTGGGTGAGCAAAAACAGGAAG
TTTTGAGAGTTCCTAGAATGGCGACAACTCTAGGTCAAGCTACATTGGGTGAGCACGTGGGTTGACTAGAAGTCGTAGAAAATGAAAGTGGTCGCAAAGACCCACTCGTTTTTGTCCTTC
F S E L I K G S N L D L E I Y G V R A G L Q D E A D K V K V L T E P H A F V P L
610 620 630 640 650 660 670 680 690 700 710 720

GCAAAATGCCGCAAAAAAGGGAATAAGGGCGACACGGAAATGTTGAATACTCATACTCTTCCTTTTTCAATCATGATTGAAGCATTTATCAGGGTTATTGTCTCATGAGCGGATACATAT
CGTTTTACGGCGTTTTTTCCCTTATTCCCGCTGTGCCTTTACAACTTATGAGTATGAGAAGGAAAAAGTTAGTACTAACTTCGTAAATAGTCCCAATAACAGAGTACTCGCCTATGTATA
C F A A F F P I L A V R F H Q I S M
AmpR promoter
730 740 750 760 770 780 790 800 810 820 830 840

TTGAATGTATTTAGAAAAATAAACAAATAGGTCATGACCAAAATCCCTTAACGTGAGTTTTCGTTCCACTGAGCGTCAGACCCCGTAGAAAAGATCAAAGGATCTTCTTGAGATCCTTTT
AACTTACATAAATCTTTTTATTTGTTTATCCAGTACTGGTTTTAGGGAATTGCACTCAAAAGCAAGGTGACTCGCAGTCTGGGGCATCTTTTCTAGTTTCCTAGAAGAACTCTAGGAAAA
ori
850 860 870 880 890 900 910 920 930 940 950 960

TTTCTGCGCGTAATCTGCTGCTTGCAAACAAAAAAACCACCGCTACCAGCGGTGGTTTGTTTGCCGGATCAAGAGCTACCAACTCTTTTTCCGAAGGTAACTGGCTTCAGCAGAGCGCAG
AAAGACGCGCATTAGACGACGAACGTTTGTTTTTTTGGTGGCGATGGTCGCCACCAAACAAACGGCCTAGTTCTCGATGGTTGAGAAAAAGGCTTCCATTGACCGAAGTCGTCTCGCGTC
970 980 990 1000 1010 1020 1030 1040 1050 1060 1070 1080

ATACCAAATACTGTCCTTCTAGTGTAGCCGTAGTTAGGCCACCACTTCAAGAACTCTGTAGCACCGCCTACATACCTCGCTCTGCTAATCCTGTTACCAGTGGCTGCTGCCAGTGGCGAT
TATGGTTTATGACAGGAAGATCACATCGGCATCAATCCGGTGGTGAAGTTCTTGAGACATCGTGGCGGATGTATGGAGCGAGACGATTAGGACAATGGTCACCGACGACGGTCACCGCTA
1090 1100 1110 1120 1130 1140 1150 1160 1170 1180 1190 1200

GenScript
Make Research Easy

AAGTCGTGTCTTACCGGGTTGGACTCAAGACGATAGTTACCGGATAAGGCGCAGCGGTCGGGCTGAACGGGGGGTTCGTGCACACAGCCCAGCTTGGAGCGAACGACCTACACCGAACTG

TTCAGCACAGAATGGCCCAACCTGAGTTCTGCTATCAATGGCCTATTCCGCGTCGCCAGCCCGACTTGCCCCCCAAGCACGTGTGTCGGGTCGAACCTCGCTTGCTGGATGTGGCTTGAC

10 20 30 40 50 60 70 80 90 100 110 120

AGATACCTACAGCGTGAGCTATGAGAAAGCGCCACGCTTCCCGAAGGGAGAAAGGCGGACAGGTATCCGGTAAGCGGCAGGGTCGGAACAGGAGAGCGCACGAGGGAGCTTCCAGGGGGA

TCTATGGATGTCGCACTCGATACTCTTTCGCGGTGCGAAGGGCTTCCCTCTTTCCGCCTGTCCATAGGCCATTCGCCGTCCCAGCCTTGTCCTCTCGCGTGCTCCCTCGAAGGTCCCCCT

130 140 150 160 170 180 190 200 210 220 230 240

AACGCCTGGTATCTTTATAGTCCTGTCGGGTTTCGCCACCTCTGACTTGAGCGTCGATTTTTGTGATGCTCGTCAGGGGGGCGGAGCCTATGGAAAAACGCCAGCAACGCGGCCTTTTTA

TTGCGGACCATAGAAATATCAGGACAGCCCAAAGCGGTGGAGACTGAACTCGCAGCTAAAAACACTACGAGCAGTCCCCCCGCCTCGGATACCTTTTTGCGGTCGTTGCGCCGGAAAAAT

250 260 270 280 290 300 310 320 330 340 350 360

CGGTTCCTGGCCTTTTGCTGGCCTTTTGCTCACATGTTCTTTCCTGCGTTATCCCCTGATTCTGTGGATAACCGTATTACCGCCTTTGAGTGAGCTGATACCGCTCGCCGCAGCCGAACG

GCCAAGGACCGGAAAACGACCGGAAAACGAGTGTACAAGAAAGGACGCAATAGGGGACTAAGACACCTATTGGCATAATGGCGGAAACTCACTCGACTATGGCGAGCGGCGTCGGCTTGC

370 380 390 400 410 420 430 440 450 460 470 480

ACCGAGCGCAGCGAGTCAGTGAGCGAGGAAGCGGAAGAGCGCCTGATGCGGTATTTTCTCCTTACGCATCTGTGCGGTATTTCACACCGCATATATGGTGCACTCTCAGTACAATCTGCT

TGGCTCGCGTCGCTCAGTCACTCGCTCCTTCGCCTTCTCGCGGACTACGCCATAAAAGAGGAATGCGTAGACACGCCATAAAGTGTGGCGTATATACCACGTGAGAGTCATGTTAGACGA

490 500 510 520 530 540 550 560 570 580 590 600

CTGATGCCGCATAGTTAAGCCAGTATACACTCCGCTATCGCTACGTGACTGGGTCATGGCTGCGCCCCGACACCCGCCAACACCCGCTGACGCGCCCTGACGGGCTTGTCTGCTCCCGGC

GACTACGGCGTATCAATTCGGTCATATGTGAGGCGATAGCGATGCACTGACCCAGTACCGACGCGGGGCTGTGGGCGGTTGTGGGCGACTGCGCGGGACTGCCCGAACAGACGAGGGCCG

610 620 630 640 650 660 670 680 690 700 710 720

ATCCGCTTACAGACAAGCTGTGACCGTCTCCGGGAGCTGCATGTGTCAGAGGTTTTCACCGTCATCACCGAAACGCGCGAGGCAGCTGCGGTAAAGCTCATCAGCGTGGTCGTGAAGCGA

TAGGCGAATGTCTGTTCGACACTGGCAGAGGCCCTCGACGTACACAGTCTCCAAAAGTGGCAGTAGTGGCTTTGCGCGCTCCGTCGACGCCATTTCGAGTAGTCGCACCAGCACTTCGCT

* L N E G D D G F R A L C S R Y L E D A H D H L S

rop

730 740 750 760 770 780 790 800 810 820 830 840

TTCACAGATGTCTGCCTGTTCATCCGCGTCCAGCTCGTTGAGTTTCTCCAGAAGCGTTAATGTCTGGCTTCTGATAAAGCGGGCCATGTTAAGGGCGGTTTTTTCCTGTTTGGTCACTGA

AAGTGTCTACAGACGGACAAGTAGGCGCAGGTCGAGCAACTCAAAGAGGTCTTCGCAATTACAGACCGAAGACTATTTCGCCCGGTACAATTCCCGCCAAAAAAGGACAAACCAGTGACT

E C I D A Q E D A D L E N L K E L L T L T Q S R I F R A M N L A T K E Q K T V

850 860 870 880 890 900 910 920 930 940 950 960

TGCCTCCGTGTAAGGGGGATTTCTGTTCATGGGGGTAATGATACCGATGAAACGAGAGAGGATGCTCACGATACGGGTTACTGATGATGAACATGCCCGGTTACTGGAACGTTGTGAGGG

ACGGAGGCACATTCCCCCTAAAGACAAGTACCCCCATTACTATGGCTACTTTGCTCTCTCCTACGAGTGCTATGCCCAATGACTACTACTTGTACGGGCCAATGACCTTGCAACACTCCC

970 980 990 1000 1010 1020 1030 1040 1050 1060 1070 1080

TAAACAACTGGCGGTATGGATGCGGCGGGACCAGAGAAAAATCACTCAGGGTCAATGCCAGCGCTTCGTTAATACAGATGTAGGTGTTCCACAGGGTAGCCAGCAGCATCCTGCGATGCA

ATTTGTTGACCGCCATACCTACGCCGCCCTGGTCTCTTTTTAGTGAGTCCCAGTTACGGTCGCGAAGCAATTATGTCTACATCCACAAGGTGTCCCATCGGTCGTCGTAGGACGCTACGT

1090 1100 1110 1120 1130 1140 1150 1160 1170 1180 1190 1200

GATCCGGAACATAATGGTGCAGGGCGCTGACTTCCGCGTTTCCAGACTTTACGAAACACGGAAACCGAAGACCATTCATGTTGTTGCTCAGGTCGCAGACGTTTTGCAGCAGCAGTCGCT

CTAGGCCTTGTATTACCACGTCCCGCGACTGAAGGCGCAAAGGTCTGAAATGCTTTGTGCCTTTGGCTTCTGGTAAGTACAACAACGAGTCCAGCGTCTGCAAAACGTCGTCGTCAGCGA

1210 1220 1230 1240 1250 1260 1270 1280 1290 1300 1310 1320

TCACGTTCGCTCGCGTATCGGTGATTCATTCTGCTAACCAGTAAGGCAACCCCGCCAGCCTAGCCGGGTCCTCAACGACAGGAGCACGATCATGCTAGTCATGCCCCGCGCCCACCGGAA

AGTGCAAGCGAGCGCATAGCCACTAAGTAAGACGATTGGTCATTCCGTTGGGGCGGTCGGATCGGCCCAGGAGTTGCTGTCCTCGTGCTAGTACGATCAGTACGGGGCGCGGGTGGCCTT

1330 1340 1350 1360 1370 1380 1390 1400 1410 1420 1430 1440

GGAGCTGACTGGGTTGAAGGCTCTCAAGGGCATCGGTCGAGATCCCGGTGCCTAATGAGTGAGCTAACTTACATTAATTGCGTTGCGCTCACTGCCCGCTTTCCAGTCGGGAAACCTGTC
CCTCGACTGACCCAACTTCCGAGAGTTCCCGTAGCCAGCTCTAGGGCCACGGATTACTCACTCGATTGAATGTAATTAACGCAACGCGAGTGACGGGCGAAAGGTCAGCCCTTTGGACAG
* Q G S E L R S V Q R
lacI
10 20 30 40 50 60 70 80 90 100 110 120

GTGCCAGCTGCATTAATGAATCGGCCAACGCGCGGGGAGAGGCGGTTTGCGTATTGGGCGCCAGGGTGGTTTTTCTTTTCACCAGTGAGACGGGCAACAGCTGATTGCCCTTCACCGCCT
CACGGTCGACGTAATTACTTAGCCGGTTGCGCGCCCCTCTCCGCCAAACGCATAACCCGCGGTCCCACCAAAAAGAAAAGTGGTCACTCTGCCCGTTGTCGACTAACGGGAAGTGGCGGA
A L Q M L S D A L A R P S A T Q T N P A L T T K R K V L S V P L L Q N G K V A Q
130 140 150 160 170 180 190 200 210 220 230 240

GGCCCTGAGAGAGTTGCAGCAAGCGGTCCACGCTGGTTTGCCCCAGCAGGCGAAAATCCTGTTTGATGGTGGTTAACGGCGGGATATAACATGAGCTGTCTTCGGTATCGTCGTATCCCA
CCGGGACTCTCTCAACGTCGTTCGCCAGGTGCGACCAAACGGGGTCGTCCGCTTTTAGGACAAACTACCACCAATTGCCGCCCTATATTGTACTCGACAGAAGCCATAGCAGCATAGGGT
G Q S L Q L L R D V S T Q G L L R F D Q K I T T L P P I Y C S S D E T D D Y G V
250 260 270 280 290 300 310 320 330 340 350 360

CTACCGAGATGTCCGCACCAACGCGCAGCCCGGACTCGGTAATGGCGCGCATTGCGCCCAGCGCCATCTGATCGTTGGCAACCAGCATCGCAGTGGGAACGATGCCCTCATTCAGCATTT
GATGGCTCTACAGGCGTGGTTGCGCGTCGGGCCTGAGCCATTACCGCGCGTAACGCGGGTCGCGGTAGACTAGCAACCGTTGGTCGTAGCGTCACCCTTGCTACGGGAGTAAGTCGTAAA
V S I D A G V R L G S E T I A R M A G L A M Q D N A V L M A T P V I G E N L M Q
370 380 390 400 410 420 430 440 450 460 470 480

GCATGGTTTGTTGAAAACCGGACATGGCACTCCAGTCGCCTTCCCGTTCCGCTATCGGCTGAATTTGATTGCGAGTGAGATATTTATGCCAGCCAGCCAGACGCAGACGCGCCGAGACAG
CGTACCAAACAACTTTTGGCCTGTACCGTGAGGTCAGCGGAAGGGCAAGGCGATAGCCGACTTAAACTAACGCTCACTCTATAAATACGGTCGGTCGGTCTGCGTCTGCGCGGCTCTGTC
M T Q Q F G S M A S W D G E R E A I P Q I Q N R T L Y K H W G A L R L R A S V S
490 500 510 520 530 540 550 560 570 580 590 600

AACTTAATGGGCCCGCTAACAGCGCGATTTGCTGGTGACCCAATGCGACCAGATGCTCCACGCCCAGTCGCGTACCGTCTTCATGGGAGAAAATAATACTGTTGATGGGTGTCTGGTCAG
TTGAATTACCCGGGCGATTGTCGCGCTAAACGACCACTGGGTTACGCTGGTCTACGAGGTGCGGGTCAGCGCATGGCAGAAGTACCCTCTTTTATTATGACAACTACCCACAGACCAGTC
S L P G A L L A I Q Q H G L A V L H E V G L R T G D E H S F I I S N I P T Q D S
610 620 630 640 650 660 670 680 690 700 710 720

AGACATCAAGAAATAACGCCGGAACATTAGTGCAGGCAGCTTCCACAGCAATGGCATCCTGGTCATCCAGCGGATAGTTAATGATCAGCCCACTGACGCGTTGCGCGAGAAGATTGTGCA
TCTGTAGTTCTTTATTGCGGCCTTGTAATCACGTCCGTCGAAGGTGTCGTTACCGTAGGACCAGTAGGTCGCCTATCAATTACTAGTCGGGTGACTGCGCAACGCGCTCTTCTAACACGT
V D L F L A P V N T C A A E V A I A D Q D D L P Y N I I L G S V R Q A L L N H V
730 740 750 760 770 780 790 800 810 820 830 840

CCGCCGCTTTACAGGCTTCGACGCCGCTTCGTTCTACCATCGACACCACCACGCTGGCACCCAGTTGATCGGCGCGAGATTTAATCGCCGCGACAATTTGCGACGGCGCGTGCAGGGCCA
GGCGGCGAAATGTCCGAAGCTGCGGCGAAGCAAGATGGTAGCTGTGGTGGTGCGACCGTGGGTCAACTAGCCGCGCTCTAAATTAGCGGCGCTGTTAAACGCTGCCGCGCACGTCCCGGT
A A K C A E V G S R E V M S V V V S A G L Q D A R S K I A A V I Q S P A H L A L
850 860 870 880 890 900 910 920 930 940 950 960

GACTGGAGGTGGCAACGCCAATCAGCAACGACTGTTTGCCCGCCAGTTGTTGTGCCACGCGGTTGGGAATGTAATTCAGCTCCGCCATCGCCGCTTCCACTTTTTCCCGCGTTTTCGCAG
CTGACCTCCACCGTTGCGGTTAGTCGTTGCTGACAAACGGGCGGTCAACAACACGGTGCGCCAACCCTTACATTAAGTCGAGGCGGTAGCGGCGAAGGTGAAAAAGGGCGCAAAAGCGTC
S S T A V G I L L S Q K G A L Q Q A V R N P I Y N L E A M A A E V K E R T K A S
970 980 990 1000 1010 1020 1030 1040 1050 1060 1070 1080

AAACGTGGCTGGCCTGGTTCACCACGCGGGAAACGGTCTGATAAGAGACACCGGCATACTCTGCGACATCGTATAACGTTACTGGTTTCACATTCACCACCCTGAATTGACTCTCTTCCG
TTTGCACCGACCGGACCAAGTGGTGCGCCCTTTGCCAGACTATTCTCTGTGGCCGTATGAGACGCTGTAGCATATTGCAATGACCAAAGTGTAAGTGGTGGGACTTAACTGAGAGAAGGC
V H S A Q N V V R S V T Q Y S V G A Y E A V D Y L T V P K V
lacI promoter
1090 1100 1110 1120 1130 1140 1150 1160 1170 1180 1190 1200

GGCGCTATCATGCCATACCGCGAAAGGTTTTGCGCCATTCGATGGTGTCCGGGATCTCGACGCTCTCCCTTATGCGACTCCTGCATTAGGAAGCAGCCCAGTAGTAGGTTGAGGCCGTTG
CCGCGATAGTACGGTATGGCGCTTTCCAAAACGCGGTAAGCTACCACAGGCCCTAGAGCTGCGAGAGGGAATACGCTGAGGACGTAATCCTTCGTCGGGTCATCATCCAACTCCGGCAAC

10 20 30 40 50 60 70 80 90 100 110 120

AGCACCGCCGCCGCAAGGAATGGTGCATGCAAGGAGATGGCGCCCAACAGTCCCCCGGCCACGGGGCCTGCCACCATACCCACGCCGAAACAAGCGCTCATGAGCCCGAAGTGGCGAGCC
TCGTGGCGGCGGCGTTCCTTACCACGTACGTTCCTCTACCGCGGGTTGTCAGGGGGCCGGTGCCCCGGACGGTGGTATGGGTGCGGCTTTGTTCGCGAGTACTCGGGCTTCACCGCTCGG

130 140 150 160 170 180 190 200 210 220 230 240

CGATCTTCCCCATCGGTGATGTCGGCGATATAGGCGCCAGCAACCGCACCTGTGGCGCCGGTGATGCCGGCCACGATGCGTCCGGCGTAGAGGATCGAGATCGATCTCGATCCCGCGAAA
GCTAGAAGGGGTAGCCACTACAGCCGCTATATCCGCGGTCGTTGGCGTGGACACCGCGGCCACTACGGCCGGTGCTACGCAGGCCGCATCTCCTAGCTCTAGCTAGAGCTAGGGCGCTTT

pET Upstream Primer

250 260 270 280 290 300 310 320 330 340 350 360

TTAATACGACTCACTATAGG
AATTATGCTGAGTGATATCC

T7 promoter

370 380 390 400 410 420 430 440 450 460 470 480
