# Supplementary material for: An engineered genetic circuit for lactose intolerance alleviation
Source: BMC Biol. 2021 Jul 5;19:137. doi: 10.1186/s12915-021-01070-9 (PMC8259030; doi:10.1186/s12915-021-01070-9)
Supplement: Supplementary file 5 — Additional file 5. Table S10 to S15. Detailed parameters of the methods. Table S10: Regular PCR reaction system; Table S11: Double enzyme reaction system; Table S12: Ligation system; Table S13: In-Fusion system; Table S14: Protocol for M9 minimal medium preparation; Table S15: Instrument setting of the plate reader for fluorescence measurement. [file 12915_2021_1070_MOESM5_ESM.docx]

**Table S10. Regular PCR reaction system**

| **Components (50 μL)** | **Volume(μL)** |
| --- | --- |
| PrimerStar Buffer | 10 |
| dNTPs (2.5 mM) | 4 |
| Primer-F (10 μM) | 1 |
| Primer-R (10 μM) | 1 |
| Template | 1 |
| PrimerStar | 0.5 |
| ddH2O  Annealing temperature | 32.5  58℃ |

**Table S11. Double enzyme reaction system**

| **Components (50 μL)** | **Volume (μL)** |
| --- | --- |
| 10 x Q.cut buffer | 5 |
| EcoRI | 1.5 |
| PstI | 1.5 |
| ddH2O | 1 |

(Reaction condition: 37℃ 30 ~ 40 min)

**Table S12. Ligation system**

| **Components (10 μL)** | **Volume (μL)** |
| --- | --- |
| T4 ligase | 1 |
| T4 ligase buffer | 4 |
| Linearized Vector | 0.5 |
| Insert Gene | 4.5 |

**Table S13. In-Fusion system:**

| **Components** | **Volume** |
| --- | --- |
| 5X In-Fusion HD Enzyme Premix | 2 μL |
| Linearized Vector * | 50 ~ 200 ng |
| Purified PCR segments** | 10 ~ 200 ng |
| dH2O(deionized water) | Total 10 μL |

***Notes*:** * < 10 kb: 50 ~ 100 ng, > 10 kb: 50 ~ 200 ng; ** < 0.5 kb: 10 ~ 50 ng, 0.5 kb ~ 10 kb: 50ng ~ 100ng, >10 kb: 50 ng ~ 200 ng

**Table S14. Protocol for M9 minimal medium preparation.**

| Volume | Agent | Component | Concentration |
| --- | --- | --- | --- |
| 200 mL | 5X stock | Na_2_HPO_4_ | 30 g/L |
|  |  | KH_2_PO_4_ | 15 g/L |
|  |  | NaCl | 2.5 g/L |
|  |  | NH_4_Cl | 5 g/L |
|  |  | CaCl_2_  (Optional) | 15 mg/L |
| 1 mL | MgSO_4_·7H_2_O | -- | 1 M |
| 10 mL | Carbon source | Sugar or glycerol | 20% |
| 0.1 mL | Vitamin B1 | -- | 0.5% |
| 5 mL | Casamino Acids | -- | 20% |
| Case dependent | Ampicillin | 100 μL/mL | |

***Notes*:** Filter sterilization for all micronutrients is recommended, and add sterilized water to final volume of 1 liter.

**Table S15. Instrument setting of the plate reader for fluorescence measurement.**

| Instrument | FlexStation 3 |
| --- | --- |
| Measurement Type | Endpoint |
| Read Mode | RFUs, Top read |
| Wavelength | Emission: 518nm |
|  | Excitation:485nm |
| Cutoff | Auto |
| Sensitivity | 6 flashes per well |
| PMT settings | Auto |
| Autocalibrate | On |
